# 2D Nanomaterials Toward Function‐Ready Superlubricity in Advanced Microsystems

**DOI:** 10.1002/adma.202520679

**Published:** 2026-04-10

**Authors:** Yushan Geng, Jun Yang, Yong Yang

**Affiliations:** ^1^ Department of Mechanical Engineering College of Engineering City University of Hong Kong Kowloon Hong Kong SAR China; ^2^ State Key Laboratory of Solid Lubrication Lanzhou Institute of Chemical Physics Chinese Academy of Sciences Lanzhou China; ^3^ Center of Materials Science and Optoelectronics Engineering University of Chinese Academy of Sciences Beijing China; ^4^ Department of Materials Science and Engineering College of Engineering City University of Hong Kong Kowloon Hong Kong SAR China

**Keywords:** 2D nanomaterials, microsystems, nanoscale tribology, solid lubrication, superlubric interfaces, ultralow friction

## Abstract

The rapid evolution of micro‐ and nanoelectromechanical systems defines a new frontier of intelligent miniaturization, where interfacial friction and wear emerge as the fundamental bottlenecks limiting functional reliability and energy efficiency. Two‐dimensional (2D) nanomaterials with van der Waals layered structures have redefined the pursuit of superlubricity, where the interfacial potential corrugation is minimized by lattice incommensurability, and energy dissipation is further suppressed through phonon spectrum mismatch and charge‐mediated coupling at the sliding interface. These atomistic principles manifest in two archetypes of superlubricity: structural superlubricity, governed by interfacial misalignment that suppresses atomic registry, and transformation superlubricity, arising from shear‐induced interface reconstructions that dynamically smooth energy landscapes. Together they delineate a continuum from geometric misfit‐driven to shear‐adaptive energy dissipation modulation, yet translating these idealized states into device‐scale systems remains formidable, as scaling, environmental perturbations, and integration losses readily destabilize ultralow‐friction behavior. This review outlines how advances in van der Waals assembly, hybrid nanomembranes, and field‐tunable heterostructures have established three classes of sliding‐driven microsystems: mechanical energy transfer, mechano‐electrical conversion, and smart interfacial control. Looking ahead, the convergence of artificial intelligence (AI)‐guided design, standardized tribological metrics, and scalable integration is redefining superlubricity as a predictive framework for engineering 2D nanomaterials, nanoscale tribology, solid lubrication, superlubric interfaces, ultralow frictionreliable, energy‐efficient microdevices.

## Graphical abstract

A unified framework links structural and transformation superlubricity with microsystem functions and deployment requirements. Mechanisms, device architectures, integration strategies, AI‐guided discovery, and benchmarking protocols are connected to define function‐ready superlubricity in advanced microsystems. The review highlights how ultralow friction can be translated from ideal interfaces into robust, scalable, sliding‐enabled micro‐ and nano‐device technologies.

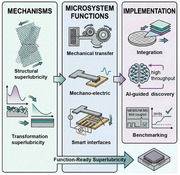

## 1 Introduction

Friction and wear are pervasive interfacial phenomena that not only consume nearly 20–30% of global primary energy but also critically limit the reliability of microdevices [1, 2, 3]. In micro‐ and nanoelectromechanical systems (MEMS/NEMS), where surface forces dominate and component dimensions approach the nanoscale, even atomic‐scale asperities can trigger severe energy dissipation, stiction, and premature failure, posing serious barriers to device miniaturization and long‐term stability [4, 5]. To overcome these challenges, superlubricity—commonly defined as a coefficient of friction (COF) below ∼0.01—has emerged as a paradigm for achieving near‐frictionless and wearless sliding in solid interfaces [6, 7]. Unlike conventional frictional motion, where energy is dissipated through phonons, electrons, and wear debris, superlubricity arises when the interfacial potential corrugation is effectively canceled, allowing energy to be stored elastically and released with strongly suppressed dissipation [8, 9, 10]. A notable case is structural superlubricity (SSL), where two atomically flat lattices in incommensurate orientations slide with nearly vanishing resistance [11, 12]. Two‐dimensional (2D) layered materials such as graphene, hexagonal boron nitrid (h‐BN), and transition‐metal dichalcogenides offer a unique platform to realize SSL, owing to their atomic smoothness and weak van der Waals interlayer coupling [4, 5, 13, 14, 15]. These breakthroughs have established 2D nanomaterials as model systems for fundamental tribophysics and as building blocks for engineering superlubric interfaces in advanced microsystems. Yet, because microsystems operate under unavoidable perturbations such as defects, contamination, and cycling, function‐ready superlubricity refers to sustaining an ultralow sliding barrier within a device‐relevant operating window with measurable durability and integration compatibility. Meeting this requirement demands not only registry‐mismatch states, but also adaptive interfacial pathways that can preserve a low sliding barrier under realistic conditions.

The concept of SSL was first rationalized by the Frenkel‐Kontorova (FK) model, which describes an atomic chain sliding over a periodic potential [7, 16]. When the lattice constants are incommensurate, the restoring forces cancel collectively, leading to vanishing static friction. This theoretical framework was later generalized to 2D lattices, providing the foundation for SSL in layered nanomaterials [17]. Experimental confirmation came from atomic force microscopy (AFM)‐based friction measurements and in situ transmission electron microscope (TEM) nanomanipulation, which demonstrated that misoriented graphene flakes can slide with COFs well below conventional limits [18, 19, 20]. Comparable behavior was subsequently observed in h‐BN and MoS_2_ [21, 22, 23], while heterogeneous interfaces showed even greater robustness due to intrinsic lattice mismatch. Beyond geometric misfit, microscopic insights revealed that SSL is reinforced by phonon spectrum mismatch across interfaces, which suppresses energy dissipation into lattice vibrations [24]. Advanced simulations, including molecular dynamics (MD) and density functional theory (DFT), further highlighted the roles of charge transfer, environmental adsorbates, and defect structures in stabilizing or disrupting ultralow‐friction states [25, 26, 27]. These results established 2D nanomaterials as archetypal SSL systems, but also emphasized their sensitivity to interfacial perturbations. Rotational torque can restore commensurability, while defects and ambient adsorbates can introduce pinning or mediator‐assisted locking that degrades superlubric sliding [28, 29, 30]. Thus, despite strong theoretical and experimental support, stabilizing SSL under realistic operating conditions remains an open challenge and motivates the search for new design and control strategies.

Extending SSL from atomically defined junctions to larger contacts has emerged as a central challenge. As contact area increases, the probability of local lattice registry rises and environmental perturbations accumulate, opening new dissipation pathways that erode ultralow‐friction states [31, 32]. To counter this, researchers have developed multiple strategies to preserve incommensurability across scales. A common route is to introduce intrinsic lattice mismatch through heterogeneous architectures, including 0D/2D nanoparticle‐membrane systems [33, 34], 2D/1D graphene‐encapsulated nanodiamond rolls [35], 2D/2D lattices such as MoS_2_/graphene and h‐BN/graphene [11, 36], and 2D/3D hybrid architectures with metals, ceramics or semiconductors [37, 38, 39]. Likewise, multiscale incommensurability arising from rotational misorientation, moiré superstructures, or polycrystalline grains can extend SSL into the micrometer regime [32, 35, 40]. By contrast, surface chemistry and environmental effects generally destabilize SSL rather than enhance it. Oxidation or fluorination of graphene increases surface corrugation and interfacial pinning, leading to higher friction and accelerated wear [41, 42, 43]. Adsorbed contaminants and ambient humidity further introduce capillary bridges or promote oxidative degradation, often causing frictional aging and elevated dissipation [44, 45, 46]. At the same time, the effect of contamination is not always irreversible. Recent results suggest that it can be history dependent, because third‐body species may reorganize during sliding and the interface can evolve toward a more weakly locked, lower‐friction state after running‐in [30]. The key issue is therefore not contamination alone, but whether the interfacial state continues to evolve under sliding. Consequently, rather than functionalization, the more effective approach is often isolation, including inert or vacuum operation, stronger interfacial adhesion to suppress delamination, and thermal or electrical cleaning to remove adsorbates [47]. These advances represent major steps toward bridging atomic‐scale SSL with larger‐scale interfaces. Yet unresolved barriers, including limited longevity under continuous sliding, poor stability in ambient environments, and difficulty integrating superlubric interfaces into device‐compatible architectures, still constrain deployment [48]. Prototype structural‐superlubric “slidevices” demonstrate that frictionless motion can in principle be scaled up [49], but they also show that practical translation requires moving beyond intrinsic friction reduction toward interfaces that perform stable device functions. Rather than serving solely as passive lubricants, 2D superlubric interfaces can act as active transduction layers in which sliding modulates mechanical and triboelectric signals. In this sense, van der Waals mechanics can be coupled with charge transfer, field tuning, and energy conversion to define a broader class of sliding‐enabled microdevices, including mechanical energy transfer units [50, 51], mechanical‐to‐electrical converters [52, 53], and field‐responsive smart interfaces [54, 55].

Building on the microscale‐to‐mesoscale advances outlined above, several milestones show that ultralow friction can be stabilized at larger contacts and under harsher conditions, although important gaps remain for MEMS/NEMS integration. Early macroscale studies on diamond‐like carbon (DLC) already suggested a transformation‐enabled route to superlow friction, in which sliding induces interfacial structural evolution, such as shear‐induced graphitization and transfer‐layer development, that lowers shear resistance [56]. These studies also revealed pronounced sensitivity to ambient adsorption and duty cycle, underscoring how difficult it is to retain ultralow friction under realistic perturbations [57]. On the macroscale, Berman et al. [40] engineered a diamond‐like carbon/graphene‐anodiamond tribofilm that sustained superlubric sliding with a COF ≈ 0.004 in dry N_2_ and near‐zero wear, demonstrating that interface‐level robustness can be achieved beyond idealized single contacts. More recently, “superlubric supersurfaces” assembled from arrays of micro‐superlubric units bridged multi‐asperity contacts and achieved COF as low as 0.006–0.007 at steel interfaces under Hertzian stress up to 1.37 GPa, evidencing a viable route from local to global slip [32]. For on‐chip proof‐of‐concepts, structural‐superlubric “slidevices” have emerged, but translating them into manufacturable MEMS demands ultrathin, conformal, patternable, and contamination‐tolerant solid lubricants. Beyond layered 2D sheets, 0D/0D metal‐MXene van‐der‐Waals nanomembranes prepared by polymer surface buckling enabled exfoliation (PSBEE) offer a new route to solid lubrication [58]. Ti‐Ti_3_C_2_T_x_ hybrid nanomembranes (<50 nm) deliver ultra‐low COF of 0.009–0.018 even under extreme AFM indentation conditions with contact pressures exceeding 10 GPa at 473 K, and, when stacked for rougher substrates, reduce macroscale COF to ∼0.08 at elevated temperature while suppressing wear, all without relying on long‐range lattice incommensurability as the sole stabilizing factor [59]. This behavior points to a complementary archetype that we term transformation superlubricity (TSL), in which shear‐driven interfacial reconstruction or structural evolution produces an easy‐shear, lower‐barrier sliding state. Its persistence under microsystem‐relevant conditions is expected to depend strongly on contamination, humidity or oxidation, and cyclic loading, which makes interfacial design and process control central to function‐ready performance. Critically, the PSBEE process, based on elasto‐capillary transfer [60], further enables wafer‐scale, conformal integration onto flat or microstructured surfaces, providing a fabrication route that is directly compatible with MEMS/NEMS integration.

**FIGURE 1:**
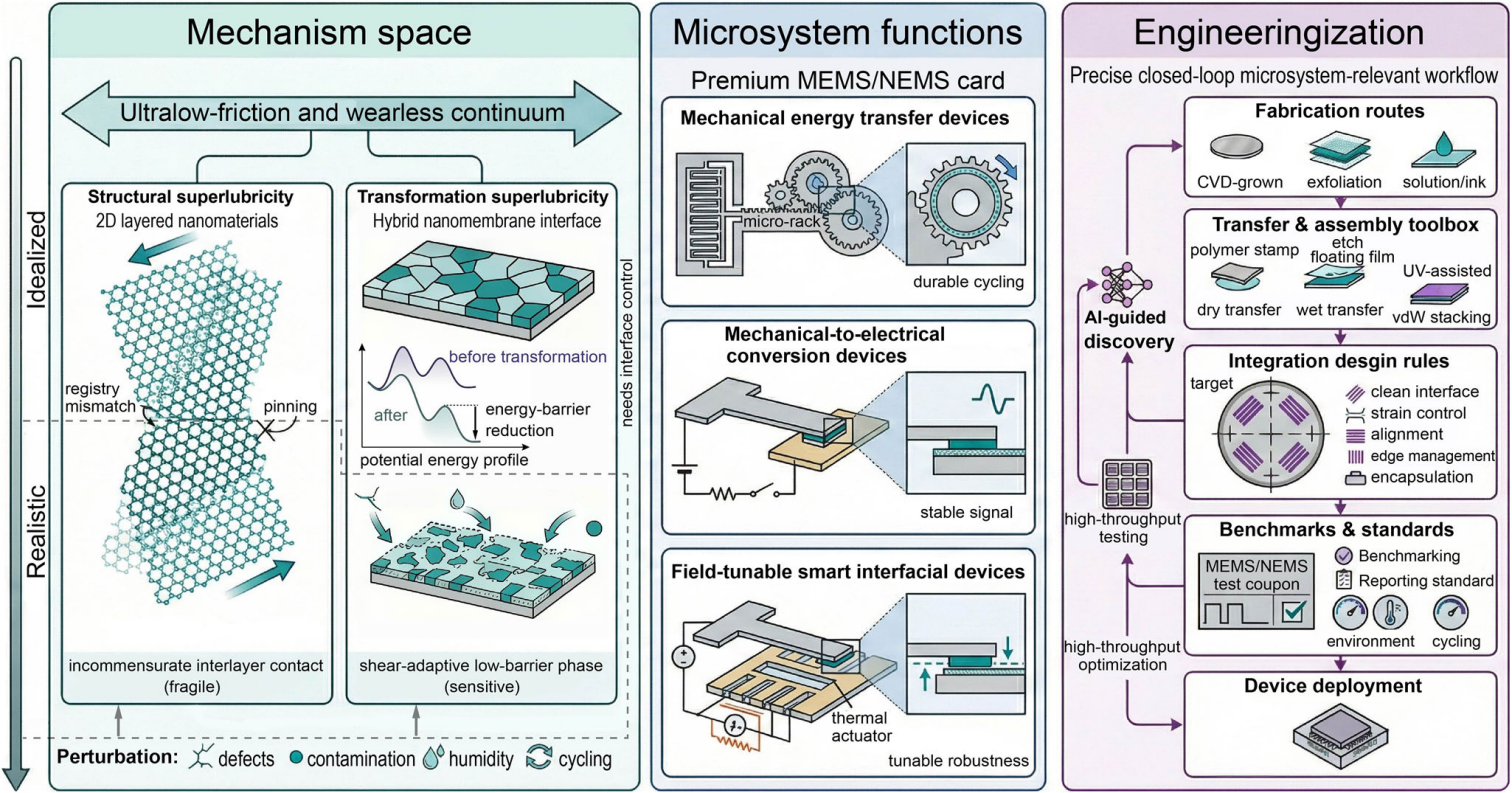
Roadmap toward function‐ready superlubricity in advanced microsystems. The framework links 2D‐enabled superlubricity mechanisms to microsystem functions and integration requirements, and outlines a pathway from interfacial physics to deployable device concepts.

As the field moves from proof‐of‐concept “slidevices” toward manufacturable microsystems, the central challenge is to connect ultralow‐friction mechanisms with device‐relevant stability under unavoidable perturbations. In this Review, we organize recent advances through a unified view that distinguishes and relates two complementary archetypes. One is SSL, in which incommensurate registry suppresses interfacial locking. The other is TSL, in which shear‐driven interfacial reconstruction or structural evolution produces a lower‐barrier, easy‐shear sliding state. This framework provides a mechanism‐level basis for explaining why ultralow friction is often fragile in idealized junctions yet can become more robust in engineered architectures that accommodate coupled mechanical and environmental perturbations. Figure 1 organizes this perspective into three connected regions spanning mechanisms, microsystem functions, and engineeringization. The first region defines the mechanistic space of structural and transformation archetypes. The second maps these archetypes onto three classes of sliding‐enabled microsystems, namely mechanical energy transfer devices, mechanical‐to‐electrical conversion devices, and field‐tunable smart interfacial devices. The third outlines the pathway to function‐ready implementation, including scalable transfer and stacking for wafer‐level integration and environmental robustness [61, 62, 63], data‐driven and physics‐informed routes for accelerated discovery and inverse design [64, 65], and standardized evaluation/reporting frameworks that connect nanoscale mechanisms to device‐level performance through interoperable metrics [66, 67]. Guided by this roadmap, we revisit the physics of ultralow friction in low‐dimensional interfaces, synthesize strategies that extend superlubricity across materials and architectures, and discuss integration and data‐enabled optimization frameworks that support function‐ready 2D superlubric microsystems.

## 2 Fundamentals of Superlubricity in Low‐Dimensional Materials

Understanding the fundamentals of superlubricity is essential for evaluating how low‐dimensional materials suppress conventional energy dissipation pathways and achieve near‐frictionless sliding. This section distills the key physical origins, experimental validations, and stability factors that determine whether such mechanisms can be translated into function‐ready lubricants for advanced microsystems.

### 2.1 Energy Dissipation and Structural Origins of Superlubricity

**FIGURE 2:**
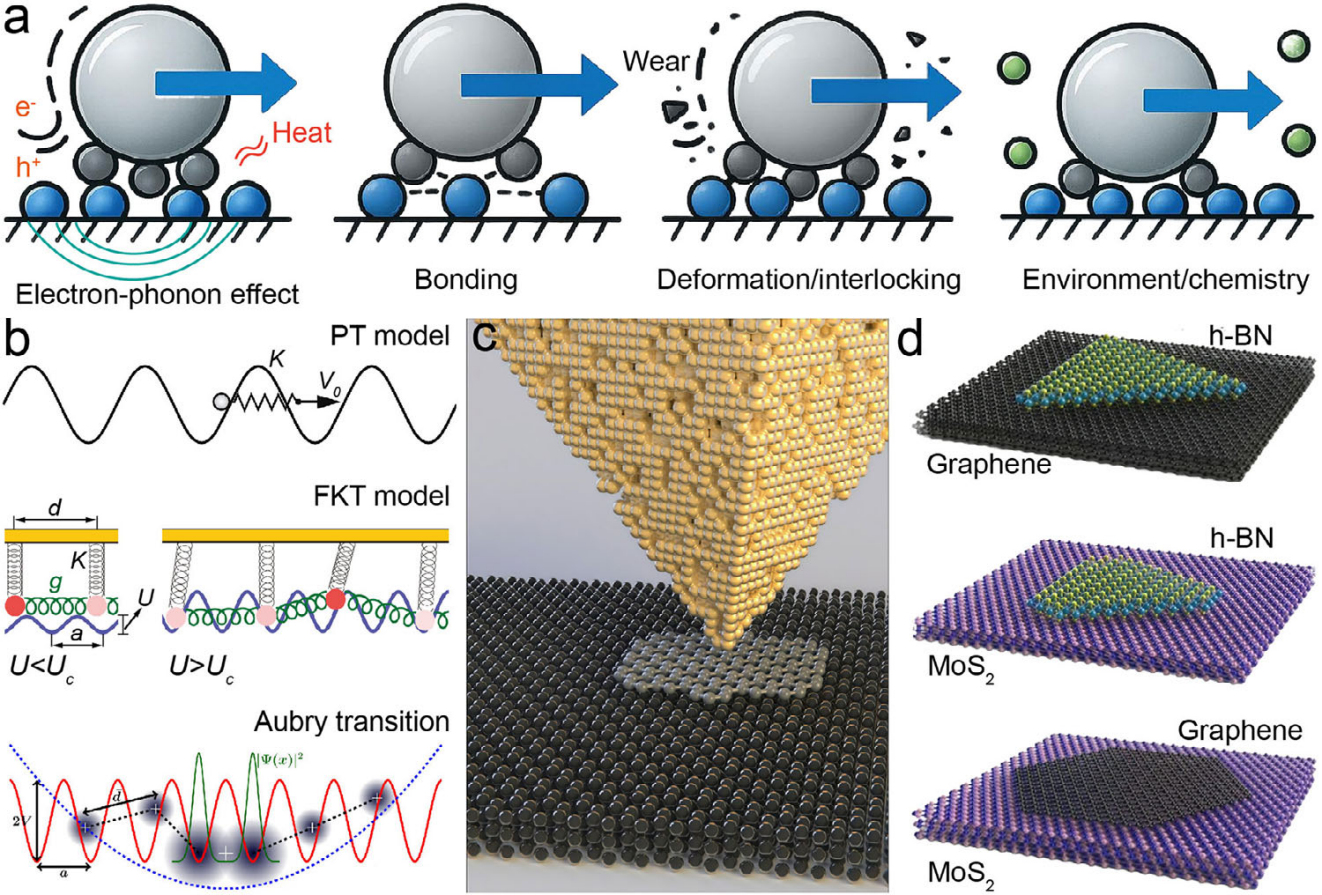
Fundamental origins and representative models of superlubricity in 2D nanomaterials. (a) Schematic illustration of possible energy dissipation mechanisms during sliding, reorganized and redrawn based on ref. [1]. Created by the authors. (b) Illustrations of the Prandtl‐Tomlinson (PT) model, the Frenkel‐Kontorova‐Tomlinson (FKT) model, and the Aubry transition, highlighting the role of commensurability in frictional instabilities. Reprinted from ref. [84, 86, 87]. with permission from Wiley‐VCH, Springer Nature and American Physical Society. (c) Schematic of AFM experiments showing a graphene flake sliding on graphite, where relative rotation of the flake reduces friction by several orders of magnitude, confirming SSL. Reproduced with permission from ref. [90]. Copyright 2018, Springer Nature. (d) Frictional characteristics of three prototypical 2D heterostructures with large lattice mismatch (graphene‐hBN, MoS_2_‐hBN, and graphene‐MoS_2_). Reproduced with permission from ref. [14]. Copyright 2022, Springer Nature.

Frictional energy dissipation in 2D interfaces can be rationalized by scaling the contact regime (Figure 2a). At small contact areas under elastic sliding, thermal activation enables atoms to hop across the corrugated interfacial potential, dissipating energy through heat [68, 69]. This potential corrugation originates from interfacial electron cloud overlap [27], where sliding induces dynamic charge transfer and static polarization, leading to charge‐density redistribution [70, 71]. Simultaneously, lattice vibrations generate phonons [72]; when vibrational modes resonate across the interface, enhanced phonon excitation increases energy loss [24], while efficient phonon transmission lowers interfacial thermal resistance [73]. As load and real contact area increase, interfacial chemical bonds form and rupture, providing discrete channels of dissipation [74]. Surface functionalization can tune bond strength, thereby controlling friction force and effective contact area [75, 76]. With further stress accumulation, lattice deformation (stretching, bending) contributes to energy loss [23, 77], and persistent shear promotes crack initiation and propagation, roughening the surface and amplifying dissipation [78, 79]. Environmental perturbations—including oxidation [41], fluorination [42, 43], adsorbate pinning [80, 81], or humidity [82, 83]—further reshape surface potential landscapes and modify these channels, explaining why ultralow friction observed in idealized experiments often deteriorates under ambient conditions.

Theoretical models have progressively captured these mechanisms (Figure 2b). The Prandtl‐Tomlinson (PT) model describes atomic stick‐slip as a particle dragged through a sinusoidal potential, introducing instability‐driven dissipation [84]. Extending to many‐body chains, the Frenkel‐Kontorova‐Tomlinson (FKT) model formalized how commensurate interfaces are pinned while incommensurate ones suppress friction [85]. The Aubry transition refined this framework by predicting a depinning phase transition: below a critical substrate corrugation, incommensurate systems become “superlubric”, as force cancellations prevent collective locking [86, 87]. Recent refinements demonstrate that the transition involves not only geometric incommensurability but also electronic redistribution and phonon spectrum mismatch, linking the microscopic potential landscape to measurable damping channels [24, 88]. Experimental observations validate these predictions. AFM studies of nanoscale graphite contacts (Figure 2c) revealed that rotating a graphene flake relative to the basal plane reduces friction by orders of magnitude [6, 12]. Such incommensurate sliding is now understood as arising from destructive phonon interference, reduced interfacial heat resistance, and dynamic charge rearrangements that minimize potential corrugation. Extending this concept, van der Waals heterostructures with large intrinsic lattice mismatch (Figure 2d), such as graphene‐hBN, MoS_2_‐hBN, and graphene‐MoS_2_ [11, 14, 36, 89], exhibit robust ultralow friction without precise angular tuning.

### 2.2 Experimental Realization of Ultralow‐Friction Across Scales

**FIGURE 3:**
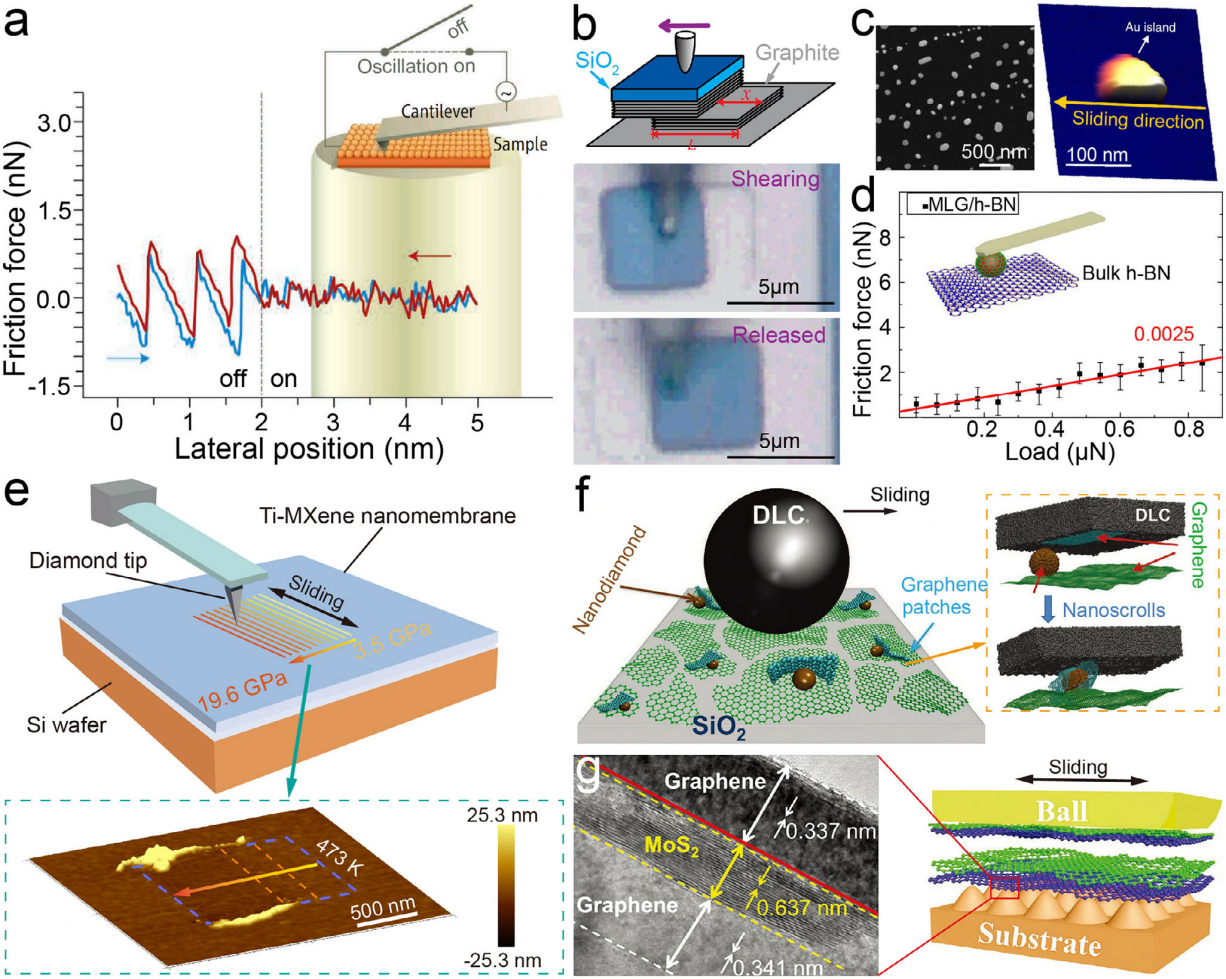
Fundamental origins and representative models of superlubricity in 2D nanomaterials. (a) Schematic illustration of atomic‐scale stick‐slip suppression through normal‐force modulation, adapted from ref. [13]. Copyright 2018, AAAS. (b) Optical images showing self‐retracting graphite flakes on HOPG, highlighting mesoscopic motion driven by ultralow interfacial friction, reproduced with permission from ref. [6]. Copyright 2012, American Physical Society. (c) SEM image and 3D AFM image of lateral manipulation of a single Au island on graphite, after ref. [46]. Copyright 2016, Springer Nature. (d) Friction‐load relationship for h‐BN sliding against SiO_2_ microspheres coated with multilayer graphene, adapted from ref. [35]. Copyright 2017, Springer Nature. (e) AFM schematic and 3D image of Ti‐MXene hybrid nanomembranes sliding against a diamond tip under wide stress ranges and elevated temperatures, image courtesy of ref. [59]. Copyright 2025, Elsevier. (f) Schematic of macroscale superlubricity achieved between DLC and graphene nanoscrolls encapsulating nanodiamonds, reproduced from ref. [40]. Copyright 2015, AAAS. (g) TEM images of layered heterostructures deposited on micropatterned steel, enabling ensemble superlubricity, reproduced with permission from ref. [32]. Copyright 2020, Wiley‐VCH.

The path toward function‐ready superlubricity has been defined by a sequence of experimental breakthroughs that progressively bridge the gap from nanometer contacts to macroscopic and device‐relevant scales. At the nanoscale, friction was shown to be actively tunable through either mechanical or electronic perturbations [13]. Socoliuc and co‐workers [91] demonstrated that applying small oscillatory normal forces to an AFM tip in contact with alkali halide substrates can suppress stick‐slip instabilities, reducing friction by over two orders of magnitude (Figure 3a). They attributed this to a modulation of the interfacial potential corrugation by dynamic changes in tip‐sample distance. Similarly, Park et al. [92] reported that bias voltages applied to the AFM tip can alter tip‐substrate interactions and modulate frictional forces, pointing to opportunities for MEMS/NEMS interfaces where integrated actuators could actively stabilize sliding. Parallel to these control strategies, the first direct observation of SSL was reported by Dienwiebel et al. [12] on highly oriented pyrolytic graphite (HOPG) and by Zettl et al. [93] on multiwall carbon nanotubes, both arising from rotationally induced incommensurability between sliding lattices. Zheng and colleagues [6, 19] further observed that micro‐scale graphite flakes on HOPG exhibit self‐retracting motion once rotated to large twist angles, where near‐incommensurate registry drives ultralow friction and interfacial energy minimization (Figure 3b). The self‐retraction velocity (*V_m_*) was shown to follow thermally activated depinning kinetics, typically described by an Arrhenius‐type relation [94] $\ln\;V_{m}=\ln V_{0}-\frac{E_{b}}{k_{B}T}$, where *E_b_* denotes the average energy barrier for depinning, *V*_0_ is a constant, *k_B_* being the Boltzmann's constant, and *T* is the absolute temperature of the sample. This mechanism offers a conceptual route for designing MEMS actuators, switches, and oscillators capable of millimeter‐per‐second motion under minimal friction. However, achieving truly near‐zero friction is hindered by material quality, edge defects, and surface contamination. More critically, such sliding is based on HOPG, which is incompatible with mainstream silicon‐based MEMS manufacturing, where high friction, strong adhesion, and brittleness of silicon cause rapid wear and debris formation.

At the microscale, efforts are devoted to overcoming the angular sensitivity and environmental fragility of SSL at interfaces by constructing heterojunctions. Van der Waals heterostructures (Figure 2c) such as graphene‐hBN [11, 36, 89], MoS_2_‐hBN [14], and fluorographene‐MoS_2_ [95] have been shown to sustain ultralow friction through lattice mismatch‐induced incommensurability. Beyond layered systems, Baykara et al. [46] demonstrated that Au(111)/graphite interfaces can repel adsorbates and exhibit structural lubricity with ultralow friction force in ambient air over ∼130 000 nm^2^ areas, breaking the long‐standing requirement of ultra‐high vacuum (Figure 3c). Nevertheless, contamination, surface oxidation, or local amorphization can introduce pinning and, in particular, mobile molecular species may mediate pseudo‐commensurate coupling that weakens structural lubricity and raises friction [96]. Importantly, this degradation is not always irreversible, because third‐body species can reorganize during sliding and partially restore a low‐friction state after running‐in [30]. To enhance the chemical resilience of heterostructures, researchers introduced moiré superlattice regions in graphene/Ge(111) interfaces [39], which suppress out‐of‐plane distortions caused by chemical modifications (e.g., fluorination or oxidation), thereby maintaining ultralow friction even after surface reactions. Nevertheless, SSL remains highly sensitive to contact stress and rotational alignment: typical AFM experiments require normal loads of only ∼10–100 nN to preserve isotropic incommensurability. In contrast, Liu et al. [35]. employed CVD‐grown multilayer graphene on SiO_2_ microspheres sliding against h‐BN, and observed COF as low as 0.0025 at local pressures exceeding 1 GPa and independent of rotational orientation (Figure 3d). Distinct from the layered 2D heterostructures discussed above, a polymer‐based manufacturing route (PSBEE) has recently enabled the creation of free‐standing, ∼35 nm Ti‐MXene hybrid nanomembranes [58], in which ductile Ti nanocrystals are chemically integrated with rigid Ti_3_C_2_T_x_ MXene nanodomains [97]. This PSBEE‐enabled 0D/0D hybrid architecture introduces mechanically dissimilar nanodomains and abundant heterointerfaces that can be reconfigured under shear. As illustrated in Figure 3e, these 0D/0D hybrids deliver COF values down to ∼0.008 under AFM indentation under contact stresses above 10 GPa and elevated temperatures (e.g. 473 K) during mesoscopic sliding, with near‐zero wear attributed to in situ oxide formation and carbon redistribution [59]. Their conformal transfer to diverse substrates demonstrates compatibility with microstructured surfaces and represents an emerging route beyond purely layered 2D sheets, though macroscale robustness remains constrained by substrate roughness and interfacial adhesion strength. Collectively, these observations support TSL in which a shear‐activated, self‐adaptive interfacial reconstruction—ere manifested as MXene decomposition coupled with oxide formation and carbon redistribution—reduces the effective sliding barrier under high stress and temperature.

A variety of tribopair combinations and lubrication strategies have been explored to extend superlubricity toward macroscopic scales. For instance, localized high‐pressure arc discharge can transform deposited MoS_2_ films into hollow, fullerene‐ or nanotube‐like nanoparticles, yielding a COF as low as 0.006 in nitrogen [98]. Hydrogenated carbon films with fullerene‐like nanostructures have also exhibited ultralow COF ≈ 0.009 under ∼20% relative humidity, although their superlubric mechanism remains debated [99]. DLC coatings were reported to reach ultralow friction via surface graphitization in dry nitrogen [57], but their performance proved extremely sensitive to atmosphere, humidity, temperature, and sliding conditions [56]. Wei et al. [100] further observed SSL by peeling the inner and outer shells of centimeter‐scale double‐walled carbon nanotubes. A milestone was achieved by Berman and co‐workers [40], who exploited spontaneous nanoscroll formation at graphene edges sliding against nanodiamond, yielding COF ≈ 0.004 under 0.5‐3 N loads (Figure 3f). Despite such breakthroughs, friction increased markedly (∼0.27) in humid air, underscoring the persistent challenge of robustness. In a subsequent extension, the same group replaced graphene with MoS_2_ in nanodiamond composites and demonstrated that in situ formation of onion‐like carbon during dry nitrogen sliding enabled stable superlubricity [101]. More recently, Chen et al. [32] deposited heterostructured layered films onto micropatterned steel surfaces. After pre‐sliding alignment, the ensemble of microcontacts collectively entered SSL states, producing ultralow friction under contact stresses of ∼0.5 GPa (Figure 3g). Despite these advances, practical deployment—especially in MEMS/NEMS—remains hampered by severe environmental sensitivity, unknown long‐term durability, and the difficulty of integrating superlubric interfaces with silicon‐based fabrication. In particular, most macroscale demonstrations still rely on finely controlled test conditions and nearly ideal materials to sustain incommensurability, limiting their technological viability.

### 2.3 Microsystem Operation Windows and Reliability Constraints

Microsystem‐ready superlubricity is constrained less by the minimum COF demonstrated in an ideal junction than by whether interfacial forces and degradation pathways remain compatible with the limited actuation and restoring forces of practical MEMS/NEMS [13]. Sliding‐enabled microdevices (e.g., actuators, positioning elements, microgears) remain difficult to commercialize because wear, seizure, and debris accumulation can rapidly terminate function. As dimensions shrink, surface forces dominate while body forces diminish, so adhesion and other interfacial forces can approach the driving and restoring forces available in typical devices [4]. Benchmarks should therefore treat adhesion and stiction as first‐order constraints and separate fabrication‐stage failure from operation‐stage failure. During release, HF removal of sacrificial SiO_2_ followed by rinse and drying can leave hydrophilic native oxide surfaces that generate strong capillary forces and “release adhesion”, which can be mitigated by eliminating the liquid‐vapor interface during drying (e.g., freeze‐drying or supercritical CO_2_ drying) or by increasing surface hydrophobicity [4]. During operation, unintended contacts induced by acceleration or electrostatic actuation can cause irreversible “in‐use stiction” once adhesion exceeds elastic restoring forces, setting an explicit reliability criterion for any molecularly thin interface intended for deployment [5]. Environmental sensitivity should be benchmarked explicitly because humidity and atmosphere history can drive intermittent friction and eventual sticking under MEMS‐representative cycling, so “function‐ready” claims should be supported by durability under realistic environmental histories rather than a single‐point COF [102]. Consistent with this framing, device‐focused assessments recommend reporting an operation window that specifies load or pressure, sliding speed, and environment, together with endurance metrics such as cycle‐to‐failure, because reliability depends on sustaining a low‐dissipation interfacial state under perturbations [49]. High‐temperature results reinforce the same logic, as a MoS_2_/graphene van der Waals heterostructure preserved structural superlubricity up to ∼850 K and maintained stable ultralow friction over >1000 cycles with minimal degradation [103]. At cryogenic temperature in vacuum, graphite nanosheets can undergo edge self‐curling and shear‐assisted rolling into ordered nanorollers, reducing friction and wear and indicating that durability can be governed by interfacial evolution toward a low‐dissipation state rather than remaining a contaminated sliding junction [104]. More broadly, sustainability‐oriented reviews emphasize that long‐term deployment requires retaining low‐friction states under high pressure, high speed, and long operating times, often through tribochemical stabilization or self‐healing tribofilm pathways rather than relying on a single ultralow‐COF datapoint [105].

A second constraint is the translation gap between nanoscale probes and device envelopes. AFM single‐asperity tests are typically performed at sliding velocities far below those in operating MEMS contacts, while the local contact pressure can reach the gigapascal regime. Probe evolution, adhesion‐controlled friction components, and tribochemistry can therefore decouple nanoscale superlubric signatures from device lifetime unless the loading and environment are mapped explicitly [106]. At the device level, wear‐limited lifetime scales strongly with the pressure‐velocity window (pv, defined as contact pressure × sliding velocity), implying that long service life at small scales often requires either operating at reduced pv or achieving fundamentally improved wear tolerance [5, 15]. For electrically functional microsystems, benchmarks must additionally capture microcontact reliability, because electrical intermittency and resistance drift can dominate failure even when friction remains low [107, 108]. The lifetime of direct‐contact radio frequency (RF) MEMS switches and microrelays is governed by metal microcontact physics and coupled degradation modes, motivating evaluation that tracks contact‐resistance stability, intermittency/noise, and failure probabilities alongside friction and wear. Hot switching is particularly demanding because it can trigger erosion through local heating, discharge, and field‐assisted processes. Experiments on platinum micro‐switches indicate that reducing environmental contamination can be more effective than lowering voltage for mitigating erosion, and that contact resistance may shift abruptly to failure after extended cycling depending on voltage and atmosphere [109]. System‐level reliability analyses further emphasize that stiction, fatigue/creep, wear, and packaging are coupled to process and materials choices, and that heterogeneous contacts or hard/soft pairing are practical strategies to stabilize conduction over cycling [110]. These considerations sharpen a central requirement for 2D‐enabled interfaces in electrically active MEMS/NEMS: low friction and low wear must be achieved together with low and stable electrical resistance. Representative studies illustrate both the trade‐off and viable routes forward, including graphitic interfaces combined with ultrathin carbon coatings for sliding electrical contacts [111] and a defect‐minimized graphite/Au van der Waals heterostructure that maintains ultralow, load‐ and speed‐stable sliding‐contact resistivity [112]. Other demonstrations highlight durability‐relevant electrical figures of merit, such as structural‐superlubric graphite microcontacts sustaining high current density while maintaining COF < 0.01 and wear‐free sliding [113] and a graphite/DLC structural‐superlubric heterojunction prototyped as a wear‐free slip‐ring contact with low contact resistivity and suppressed intermittency [111]. Accordingly, for electrically active MEMS/NEMS, a minimal benchmark set should report friction stability and cycle‐to‐failure together with electrical metrics (contact resistance mean, drift, intermittency, and contact resistivity) under specified switching mode and environment, alongside the mechanical pv window. Long‐life studies indicate that ultralow friction can be reached after running‐in and then sustained over very large cycle counts and long‐distance wearless sliding at device‐relevant speeds in controlled atmospheres, supporting endurance as a primary reporting metric [114].

Packaging and integration set the boundary conditions of the operation window, and therefore define the realistic stability envelope for deployable superlubric interfaces. Wafer‐level packaging studies show that many microsystems rely on controlled cavity pressure and hermetic sealing (often with getters) to stabilize dynamic response, and that bonding approaches must respect thermal budgets and interconnect/feedthrough requirements at wafer scale [115]. In 2D‐material roadmaps, device lifetime is recognized as extremely short without adequate protection, motivating highly hermetic yet low‐cost packaging and barrier‐layer strategies (e.g., nitride/boron nitride protection combined with wafer‐level bonding) [116]. In this context, graphene or h‐BN have been explored as ultrathin barrier encapsulation layers to limit moisture/contaminant ingress and associated drift of superlubric interfaces [117, 118, 119]. Low‐temperature heterogeneous bonding enabled by ductile 2D metallic nanosheets (e.g., Au, Cu and high‐entropy‐oxide) further supports wafer‐compatible integration under tight thermal budgets [120, 121, 122]. Because packaging‐induced stress can shift contact states over cycling, the mechanical compliance of 2D layers is also relevant in mitigating CTE‐mismatch‐driven drift that promotes stiction and wear in microscale interfaces [123]. At the system level, roadmaps emphasize that architecture choice, process compatibility, and variation control across arrays bound what can be realized at scale, so tribological targets should be specified together with integration constraints rather than appended after device fabrication [124]. Temperature and atmosphere further reshape interfacial chemistry and kinetics. At elevated temperatures, low‐friction states are often sustained by tribochemical passivation and in situ formation of low‐shear surface films, so durability depends on maintaining these protective states under continuous sliding [125]. Accordingly, microsystem benchmarking should report temperature and environmental history together with contact‐state evolution, rather than treating room‐temperature friction as directly transferable. These packaging, integration, and environment‐coupled limits align with sustainable‐superlubricity perspectives that treat durability, load‐bearing capability, and cross‐scale translation as the practical bottlenecks for moving from laboratory demonstrations to real machines.

## 3 Toward Functional Interfaces in Advanced Microsystems

Translating superlubricity from model systems to functional microsystems requires not only materials with ultralow friction, but also the integration of multiple mechanical and electronic properties under realistic conditions. In this section, we outline the property landscape of low‐dimensional materials, examine the operational benchmarks of microsystems, and review emerging strategies for fabricating and integrating complex interfaces.

### 3.1 From Intrinsic Properties to Integrated Performance of 2D Nanomaterials

First principles and empirical potential calculations consistently show that the hexagonal lattice and strong covalent bonding endow single‐layer graphene with exceptional in‐plane stiffness, with Young's modulus near 1.03 TPa [126, 127, 128] and Poisson's ratio ∼0.144 [129, 130]. Owing to its atomic thinness, graphene exhibits extremely low flexural rigidity, with a bending modulus ∼1.5 eV, manifesting as ripples, wrinkles, and folds that impart extraordinary flexibility [131]. Other layered 2D nanomaterials such as h‐BN [129], MoS_2_ [132], and phosphorene [133] display similarly linear elastic responses, though most enter a nonlinear regime when the strain exceeds ∼5% [134]. At high normal or shear loads, such nonlinearity manifests as load‐dependent stiffness and stress localization, which can trigger contact instabilities and accelerate fracture initiation. Beyond graphene, MXenes (M_n+1_X_n_T_x_, where M, X, and T denote transition metals, carbon/nitrogen, and surface terminations, respectively) have emerged as mechanically tunable 2D carbides/nitrides [135]. DFT predicts elastic constants above 500 GPa for M_2_X, M_3_X_2_, and M_4_X_3_ MXene sheets [136], indicating their exceptional mechanical stiffness. For instance, Ti_2_CT_x_ shows higher stiffness than MoS_2_ (∼135 GPa) but lower than h‐BN (∼310 GPa) and graphene (∼350 GPa) [137]. MD simulations further indicate a positive correlation between thickness and bending rigidity, and in the case of monolayers, the bending rigidity follows the trend: MoS_2_> Ti‐based MXene > graphene [138]. Unlike other layered 2D nanomaterials, MXenes exhibit greater tunability in their mechanical and tribological behavior owing to their diverse bonding configurations and highly variable surface terminations [139]. Guo et al. [140]. predicted that ‐O_2_ termination can significantly enhance the mechanical resilience of Ti_2_C MXene, increasing its ultimate strain under biaxial and uniaxial tensile stresses by approximately 20%, 28%, and 27%, respectively. In parallel, Zhang et al. [141]. demonstrated that ‐O_2_ termination markedly reduces the sliding energy barrier of Ti‐based MXenes, with Ti_2_CO_2_ showing a barrier as low as ∼0.017 eV, approaching the ultralow value of graphene (∼0.002 eV), thereby suggesting strong potential for SSL applications. Experimentally, AFM nanoindentation [142] and MEMS tensile tests [143] confirm these theoretical trends. Nearly perfect monolayers reach breaking strengths of tens to >100 GPa [144], with graphene measured at ∼130 GPa [142] and MoS_2_ at ∼27 GPa [145].

**FIGURE 4:**
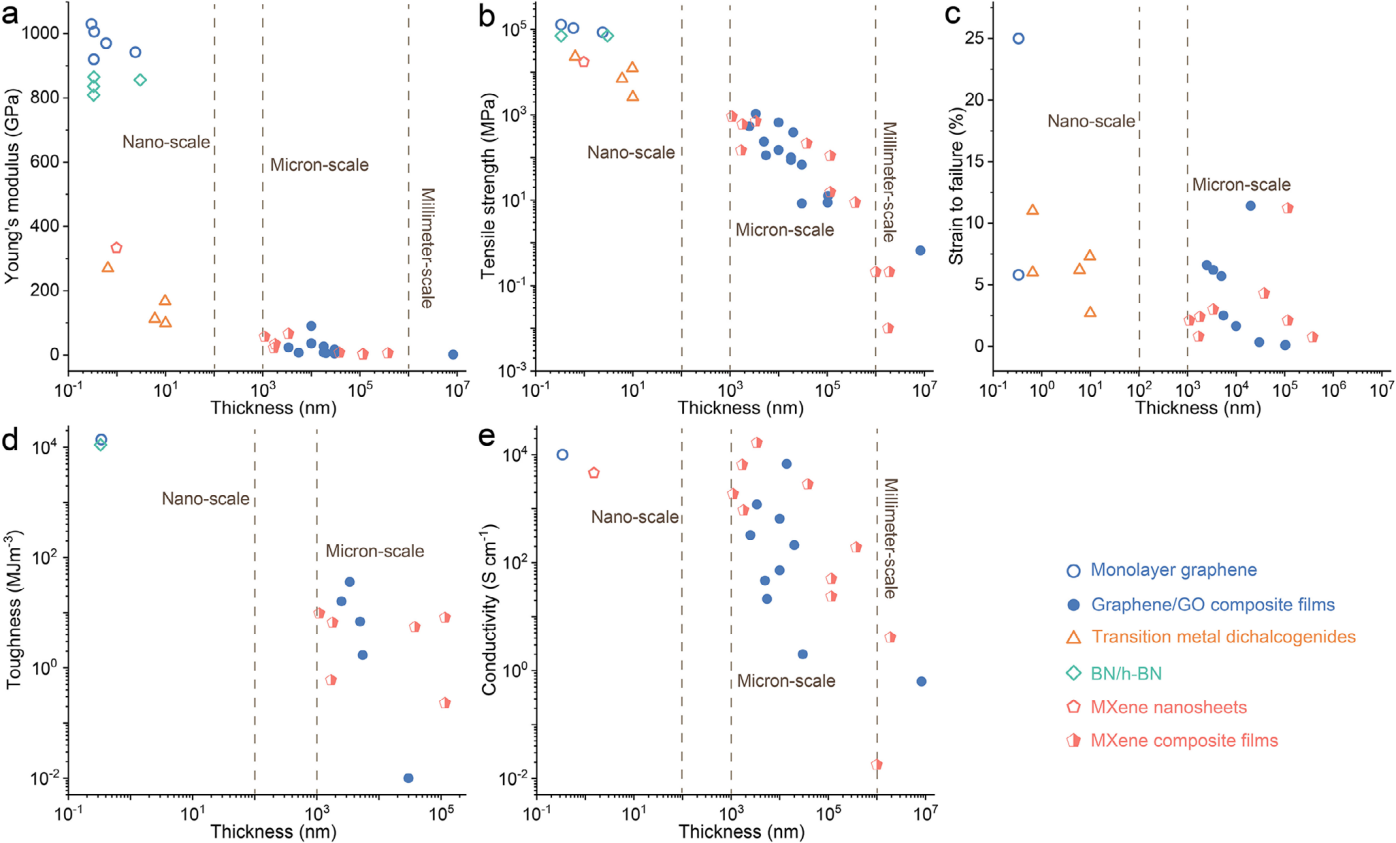
Thickness‐dependent mechanical and electrical properties of representative 2D nanomaterials. (a) Young's modulus, (b) tensile strength, (c) strain‐to‐failure, (d) toughness, and (e) electrical conductivity are compared across monolayers, assembled micron‐scale films, and bulk‐scale laminates, highlighting the degradation of intrinsic properties upon upscaling. Detailed references and numerical values are summarized in Table 1.

Equally critical for microsystems is electronic functionality. Theoretically, graphene's Dirac dispersion yields ultrahigh carrier mobility (>200,000 cm^2^/Vs) [146] and electrical conductivity (up to 10^4^ S cm^−1^) [147, 148], while h‐BN provides wide‐bandgap insulation (5.9 eV) [149], and MoS_2_ [150, 151] or black phosphorus [152] supply semiconducting bandgaps suitable for field‐effect devices. Junctions such as graphene/h‐BN or MoS_2_/graphene exhibit modified charge transfer and interfacial states [153], directly influencing frictional dissipation via electron‐phonon coupling. T_3_C_2_T_x_ MXenes combine good conductivity (4600 ± 1100 S cm^−1^) with tunable terminations [154], allowing dynamic adjustment of charge density and thus interfacial energy landscapes. However, once 2D crystals are assembled into micrometer‐thick functional films by techniques such as vacuum filtration [155], wet spinning [156], electropolymerization [157], electrodeposition [158], blade coating [159], or confined evaporation [160], their strengths plummet to merely MPa‐hundreds of MPa, conductivity drops to within a few hundred S cm^−1^ and capacitance reduces to tens–hundreds F g^−^^1^. To counteract this degradation induced by thickness increase, extensive efforts have been devoted to composite design and process optimization, aiming to recover mechanical robustness and electrical functionality (as summarized in Table 1 and Figure 4). The Young's modulus of ideal monolayer graphene or h‐BN exceeds 800 GPa within sub‐nanometer thickness, yet declines sharply to ∼200 GPa at a few nanometers (Figure 4a). Once integrated into micrometer‐thick graphene oxide (GO) or MXene‐based composite films, even with metal‐ion doping or interfacial bonding reinforcements, the modulus further decreases below 100 GPa and approaches only ∼1 GPa at millimeter scale. Figure 4b,c further illustrates thickness‐dependent tensile behaviors: monolayer graphene exhibits ultimate strength up to 130 GPa and ∼25% strain, whereas most other 2D crystals remain below 12% strain; with increasing thickness to ∼10 nm, strength rapidly decreases to ∼20 GPa, and once at micro‐millimeter levels, graphene‐ and MXene‐based films weaken from ∼1 GPa to < 0.01 MPa, with fracture strains generally below 5%. Figure 4d highlights the same trend in toughness: single‐layer graphene and h‐BN exhibit extreme values near 10^4^ MJ m^−3^, while bulk‐scale composite films drop precipitously into the 10^−2^–10^1^ MJ m^−3^ range. In contrast, the electrical response shows a partially different scaling (Figure 4e): although many micrometer‐thick graphene and MXene composites surprisingly maintain high conductivities of 10^3^–10^4^ S cm^−1^ comparable to their nanoscale counterparts, a further increase to millimeter thickness reduces conductivity to below 1 S cm^−1^. This degradation arises from poor alignment, porosity, and weak interfacial bonding, all of which compromise stress transfer and carrier transport. This interplay of mechanical and electronic degradation highlights why scalable integration remains a bottleneck.

**TABLE 1:** Mechanical and electrical properties of selected 2D nanomaterials at different length scales, from monolayer to macroscopic assemblies.

Materials	Prediction/preparation	Thickness	Young's modulus (GPa)	Tensile strength (MPa)	Strain to failure (%)	Toughness (MJm^−3^)	Conductivity (S cm^−1^)	Refs.
Graphene	DFT	0.299 nm	1030	—	—	—	—	[129]
Graphene	Mechanical exfoliation	0.598/2.392 nm	970/942	107.7/85.3 GPa	—	—	—	[161]
Graphene	Mechanical exfoliation	0.335 nm	1000	130 GPa	25	—	—	[142]
Graphene	Chemical vapor deposition	0.335 nm	920	53 GPa	5.8	—	—	[162]
Graphene	Arc‐discharge method	0.34 nm	1006	—	—	13 800	—	[163]
BN	Induction heating	0.33 nm	836	—	—	11 100	—	[163]
BN	Mechanical exfoliation	0.334/3.006 nm	865/856	70.5/71.0 GPa	—	—	—	[161]
Graphene	Chemical vapor deposition	0.34 nm	—	—	—	—	10 000	[147]
MoS_2_	Mechanical exfoliation	0.65 nm	270	23 GPa	6–11	—	—	[145]
MXene	Chemical etching	0.98	333	17.3 GPa	—	—	—	[164]
MXene nanosheet	Chemical etching	1.5 nm	—	—	—	—	4600	[154]
h‐BN	DFT	0.33 nm	809	—	—	—	—	[129]
MoTe_2_	Chemical vapor transport	6/10 nm	112.5/99	7/2.6 GPa	6.2/2.7	—	—	[165]
WSe_2_	Chemical vapor transport	9.8 nm	167.3	12.4 GPa	7.3	—	—	[166]
MXene film	Chemical etching	1.7 µm	21.5	146	0.8	0.6	6512	[167]
MXene‐Ga^3+^‐cellulose film	Blade coating	1.8/1.1 µm	33.6/56.6	598.1/908.4	2.4/2.1	6.6/9.7	936/1875	[84]
MXene film	Evaporation	3.4 µm	66	707	3	—	16 600	[160]
π‐bridged graphene film	Vacuum filtration	3.4 µm	23.3	1054	6.2	36	1192	[168]
Graphene‐AP‐DSS film	Hummers’ method	∼2.5 µm	—	538.8	6.6	16.1	321.9	[169]
GO‐PAAP‐Eu^3+^	Gel‐casting method	5‐6 µm	6.9	112.1	2.5	1.7	21.2	[170]
Graphene‐MoS_2_ film	Vacuum filtration	∼5 µm	—	235.3	∼5.7	6.9	46.4	[171]
Graphene film	Hummers’ method	10 µm	35	—	—	—	72	[172]
GO film	Continuous centrifugal casting	10 µm	90	660	—	—	650	[173]
Graphene film	Compression	14 µm	—	—	—	—	6740	[174]
GO wire	Wet spinning	20 µm	5.3	385.7	11.4	—	210.7	[156]
GO film	Hummers’ method	10–30 µm	22.2/36.4/16.6	67.5/82.2/149.4	0.34/0.25/1.65	—	—	[175]
GO‐Mg^2+^/Al^3+^	Hummers’ method	18 µm	7.6/26.2	86.9/100.5	—	—	—	[176]
Graphene film	Hummers’ method	30 µm	4.2	8.4	—	0.01	2	[177]
MXene‐cellulose	Vacuum filtration	38 µm	7	212	4.3	5.5	2837	[178]
Graphene paper	Electropolymerization	102 µm	—	8.8	0.08	—	—	[179]
Graphene‐Polyaniline paper	Electropolymerization	104 µm	—	12.6	0.11	—	—	[179]
MXene‐cellulose	Vacuum filtration	116 µm	3.2/1.6	109.6/15.4	11.2/2.1	8.1/0.23	23.5/50.2	[180]
Foamed MXene film	Chemical etching	376 µm	5.4	8.8	0.75	—	192	[181]
MXene/CNF aerogel	Mechanical grinding	1 mm	—	0.21	—	—	0.018	[182]
MXene‐carbon foam	Chemical etching	1.8 mm	—	0.01	—	—	—	[183]
MXene‐Aramid nanofiber	Chemical etching	1.9 mm	—	0.21	—	—	4.1/48.3	[184]
GO aerogel	Supercritical CO_2_ drying	8.3 mm	1.2/6.2 MPa	0.04/0.66	—	—	0.63	[185]

**FIGURE 5:**
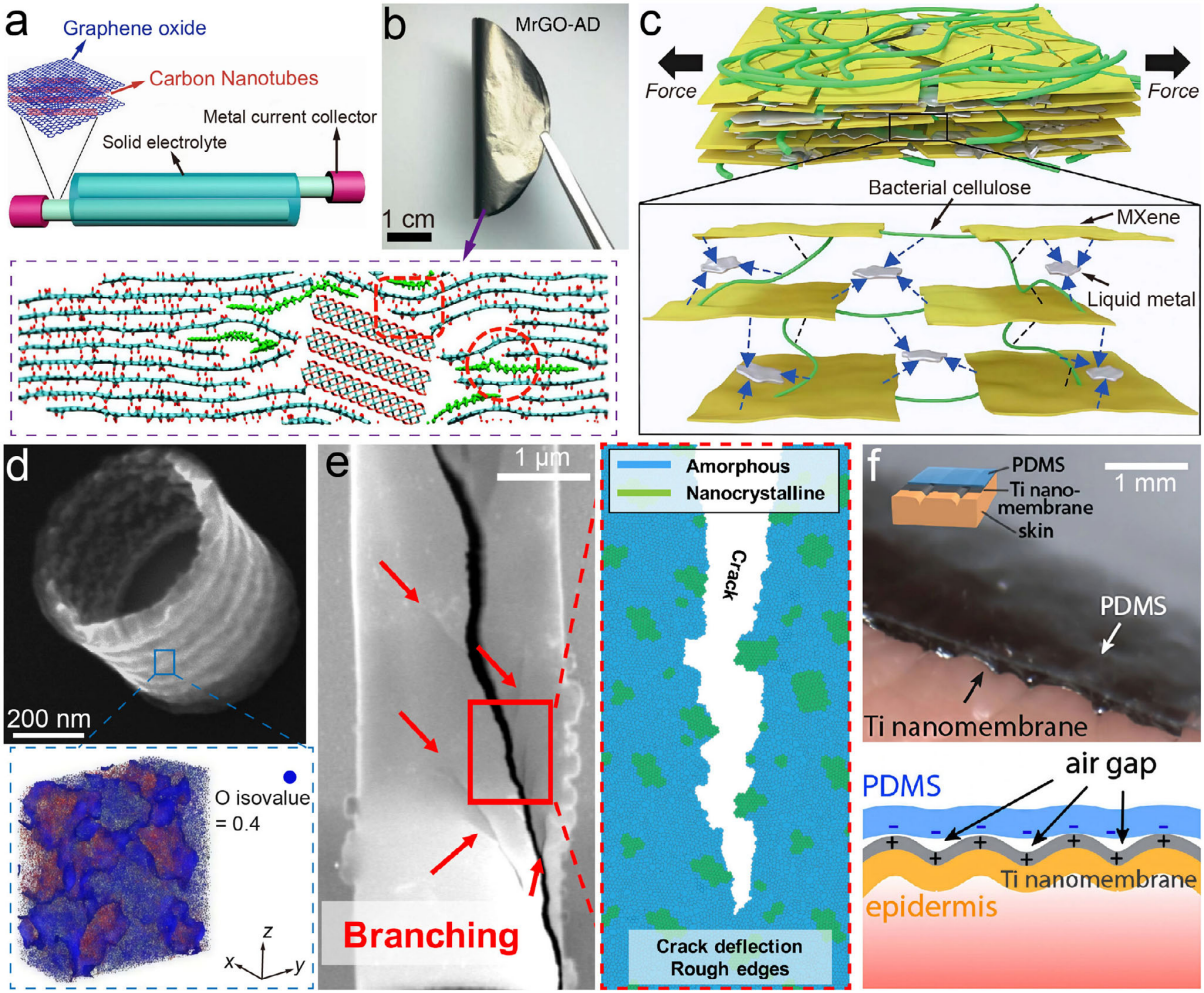
Strategies for reinforcing 2D nanomaterials. (a) Schematic of wire‐shaped supercapacitors fabricated from graphene oxide reinforced with few‐walled carbon nanotubes. Adapted from ref. [156]. Copyright 2015, American Chemical Society. (b) Photograph and atomic model of foldable graphene oxide platelets functionalized with MXene nanosheets. Image reproduced from ref. [177]. Copyright 2020, Springer Nature. (c) Schematic illustration of MXene nanosheets sequentially bridged by liquid metal and bacterial cellulose, highlighting hydrogen and coordination bonds that strengthen interfacial interactions. Figure adapted from ref. [167]. Copyright 2024, AAAS. (d) SEM image and 3D elemental mapping of Zr_55_Cu_30_Al_10_Ni_5_ metallic glass nanotubes, showing their characteristic oxide scaffold. Data reproduced from ref. [189]. Copyright 2024, Springer Nature. (e) Fracture SEM image and schematic of the toughening mechanisms in single‐layer amorphous carbon nanocomposites. Figure taken from ref. [143]. Copyright 2025, Cell Press. (f) Photograph and schematic of a Ti‐based nanomembrane used as a single‐electrode triboelectric nanogenerator, illustrating its mechanical‐to‐electrical response. Adapted from ref. [97]. Copyright 2023, American Chemical Society.

To overcome these limitations, many strategies focus on composite reinforcement and structural engineering. For example, Ma et al. [156] dispersed few‐walled carbon nanotubes into GO to fabricate ∼20 µm‐diameter fibers (Figure 5a), which overcame the rapid drop of strength and conductivity with length in linear graphene devices. Similarly, Chen et al. [186] introduced cellulose nanocrystals into GO to yield fibers with cross sectional areas of 792–914 µm^2^ and strengths around 200 MPa, while maintaining nearly unchanged capacitance after 500 bending cycles. Beyond carbon‐based hybrids, Zhou et al. functionalized GO sheets with T_3_C_2_T_x_ MXene (Figure 5b), where Ti‐O‐C covalent bonding and MXene interlayer sliding increased tensile strength from 82 to 700 MPa while maintaining high toughness (42.7 MJ m^−3^) and conductivity (1329 S cm^−1^). At the thin‐film scale, processing routes such as enhancing orientation [159], strengthening interfacial interactions [187], and reducing porosity [188] are key to improving stress transfer efficiency and multifunctionality. Along these lines, Li et al. [167] incorporated liquid metal Ga and bacterial cellulose into MXene films through layer‐by‐layer blade coating (Figure 5c), sequentially bridging hydrogen and coordination bonds to push strength to 908 MPa, modulus to 65 GPa, and conductivity to 1875 S cm^−1^. Meanwhile, nanoscale design strategies provide further insights into potential strengthening mechanisms for functional 2D nanomaterials. Yang et al. [189] fabricated metallic glass nanotubes with an oxide scaffold (Figure 5d), achieving ∼14% recoverable strain through deformation‐driven bond reconfiguration. Lou et al. [143] showed that dispersed nanocrystals in 2D amorphous carbon enhanced fracture resistance eightfold relative to graphene (Figure 5e), through mechanisms of crack blunting, deflection, and bridging. Park et al. [97] further reported hybrid nanomembranes composed of Ti_3_C_2_T_x_, Ti, and TiO_2_ nanocrystals, exhibiting ∼6 GPa strength and ∼30% ductility, which could function as skin‐mounted triboelectric nanogenerators with stable mechano‐electrical outputs (Figure 5f). Altogether, 2D nanomaterials intrinsically possess extraordinary strength, flexibility, and tunable electronic behavior, but their transition into integrated films often causes drastic deterioration, undermining their tribological and functional reliability. The central scientific challenge is to preserve intrinsic mechanical and electronic advantages while ensuring structural integrity at device scale. Composite strategies and nanoscale toughening offer promising pathways, yet realizing multifunctional films that simultaneously deliver high modulus, toughness, conductivity, and low friction under realistic loads remains the critical frontier.

### 3.2 Sliding‐Enabled 2D Interfaces for Microsystems and Devices

Interlayer sliding in 2D nanomaterials provides a universal route to translate atomic‐scale superlubricity into microsystem functionality. Depending on the direction of energy and information flow, sliding‐based devices can be grouped into three categories: (i) mechanical energy transfer devices, (ii) mechanical‐to‐electrical conversion devices, and (iii) field‐tunable smart interfacial devices. Each exploits the ultralow‐friction state in distinct ways, from dissipationless motion to charge generation and programmable coupling under external fields.

#### 3.2.1 Mechanical Energy Transfer Devices

**FIGURE 6:**
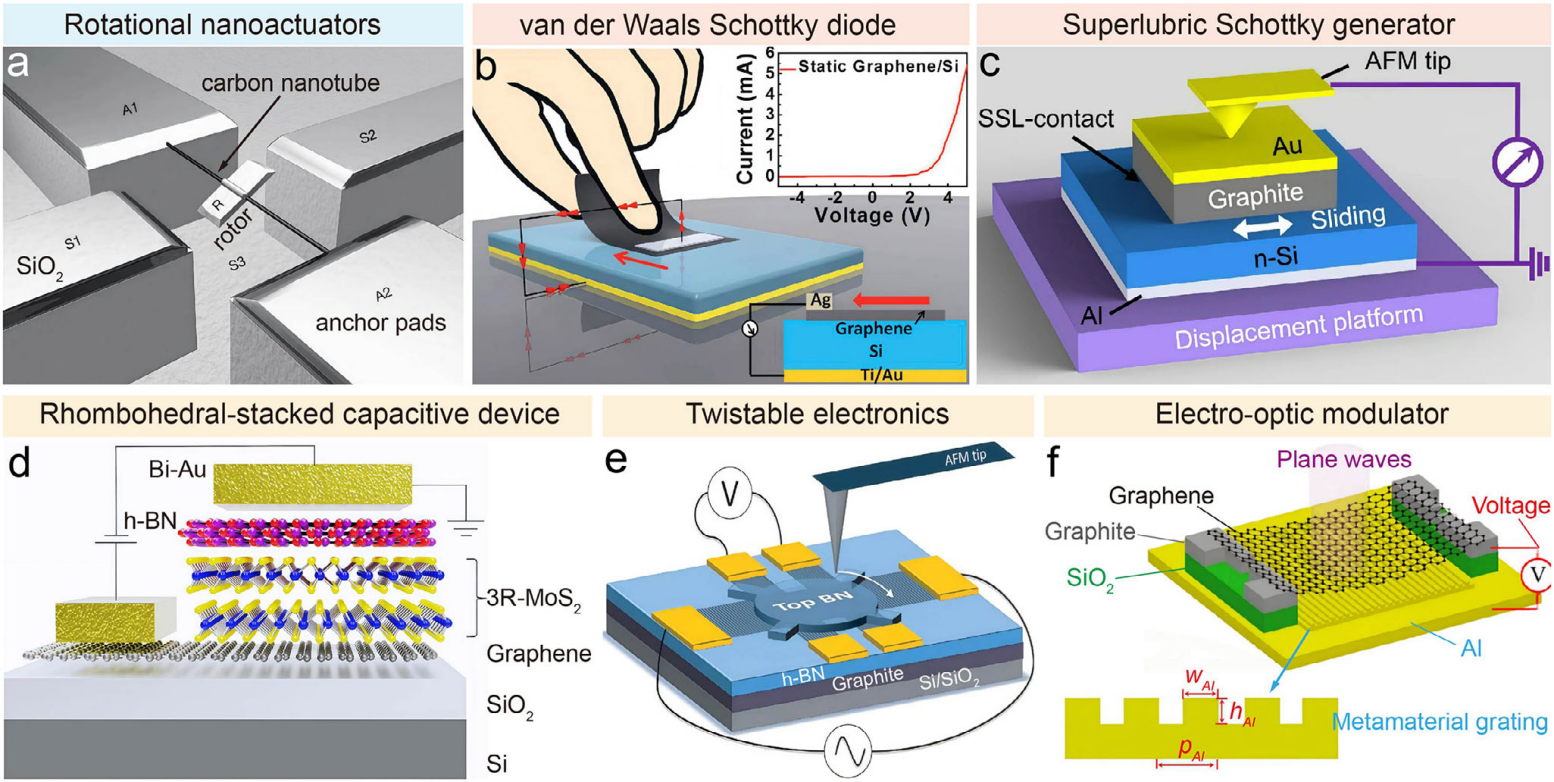
Sliding‐enabled microsystem devices based on 2D nanomaterials. (a) Conceptual illustration of a nanoactuator using a multiwalled carbon nanotube as a freely rotating shaft. Adapted from ref. [50]. Copyright 2003, Springer Nature. (b) Schematic of a van der Waals moving Schottky diode; inset: current‐voltage curve from a graphene film/Si heterostructure generator. Reproduced with permission from ref. [52]. Copyright 2018, Wiley‐VCH. (c) Graphite/n‐Si Schottky superlubric generator (SSL‐stabilized) based on a sliding junction. Adapted from ref. [53]. Copyright 2021, Springer Nature. (d) Schematic of a Bi/h‐BN/3R‐MoS_2_/graphene capacitive device on a SiO_2_/Si substrate. Reproduced with permission from ref. [54]. Copyright 2024, Springer Nature. (e) Twistable electronics enabled by a rotatable h‐BN/graphene heterostructure. Reprinted from ref. [200]. Copyright 2018, AAAS. (f) Schematic of a graphene–metamaterial integrated electro‐optic modulator. Reproduced with permission from ref. [203]. Copyright 2022, De Gruyter.

The earliest demonstrations of superlubric sliding in low‐dimensional systems were mechanical transducers in which motion is transmitted purely by van der Waals interfaces. In microsystems, beams or plates are usually actuated by electrostatic or electromagnetic forces to deform elastically, where the competition between driving force and interfacial resistance governs the motion speed [4]. Classic experiments by Zettl and co‐workers [93] showed that nested carbon nanotubes undergo telescopic oscillations with static and dynamic intershell friction below 10^−14^ N per atom, maintaining nanosecond recovery times—ideal for bearings, springs, and nanoscale guides. The dynamics are governed by the van der Waals potential $U_{\left(X\right)}=-\frac{4\pi \sqrt{3}D\zeta \prod }{9a^{2}}x$ (where *a* is the carbon‐carbon bond length, *D* is the diameter of the core, ζ is the dimensionless numerical factor, ∏ is the van der Waals energy defined on a single‐shell core and *x* is the separation of the centers of the core and the outer shells) [190], producing oscillation frequencies up to 1.39 GHz. The same intershell sliding underpins rotary actuation. Fennimore et al. [50] demonstrated a voltage‐driven rotational actuator based on multiwalled carbon nanotubes, where a rotor plate undergoes reproducible ∼180° switching within ∼33 ms and operates at frequencies of tens of hertz (Figure 6a). Bockrath et al. [51] further showed that intershell sliding can function as a nanoscale switch with closure times in the femtosecond‐picosecond range, comparable to chemical bond breaking processes. Toward manufacturability, Subramanian et al. [191] developed shell‐peeling and dielectrophoretic assembly routes, enabling batch‐fabricated carbon nanotube bearings compatible with silicon MEMS platforms. Graphene‐based sliding resonators also illustrate how superlubric contacts alter mechanical response. Conventional fixed‐end resonators are widely used for mass or force sensing in RF circuits, while Ying et al. [192] built a sliding‐clamped graphene resonator on a grooved SiO_2_ track. Its ultralow‐friction contact induces hysteresis loops in resonance frequency under gate‐voltage cycling [193], providing a unique platform for quantifying nanoscale frictional dissipation. More recently, MEMS‐style actuator architectures have begun to incorporate structural‐superlubric interfaces as functional sliding joints rather than as purely nanoscale demonstrations. A representative in‐plane electrostatic actuator based on a graphite/SiO_2_ structural‐superlubric contact achieved large‐stroke, controllable reciprocating motion while maintaining interfacial integrity over extended cycling, underscoring that device‐level performance depends on guided contact geometry and edge/defect management as much as on intrinsically low friction [194]. Despite these advances, scaling remains the primary challenge: mechanical property degradation, unavoidable defects, and edge effects disrupt incommensurate sliding and lead to pinning, ultimately undermining the robustness of superlubricity [195].

#### 3.2.2 Mechanical‐To‐Electrical Conversion Devices

Sliding‐induced charge transport at 2D interfaces offers a direct route to convert mechanical motion into electrical energy or signals, which is attractive for self‐powered sensing and on‐chip energy harvesting. Unlike triboelectric nanogenerators that rely on charge transfer by friction, these devices exploit the high carrier mobility and low interfacial barriers of 2D nanomaterials to dynamically form Schottky contacts during sliding. A representative example was reported by Thundat et al. [196], where a Pt/Ir‐coated AFM tip sliding on MoS_2_ generated a steady output current of ∼0.8 nA, corresponding to a current density of 10^6^ A/m^2^. Extending this concept, lateral motion of a graphene/metal thin film across a semiconductor substrate enables a van der Waals Schottky‐diode generator [52], delivering continuous direct‐current densities up to 40.0 A/m^2^ and powering a light‐emitting diode for over 10 000 operation cycles (Figure 6b). In parallel, superlubric Schottky generators explicitly leverage SSL to stabilize the sliding junction and suppress wear‐related parasitic dissipation (Figure 6c), enabling a clearer attribution of the output to periodic modulation of the Schottky barrier and depletion region at the 2D/semiconductor interface [53]. Together, these devices illustrate complementary routes for mechanical‐to‐electrical conversion based on sliding Schottky junctions, spanning van der Waals contact‐based rectification and structural‐superlubricity‐stabilized interfacial performance. However, practical implementation faces two key bottlenecks: first, graphene and related materials often exhibit high contact resistance and limited load‐bearing capacity under large‐area sliding, necessitating metal buffer layers (e.g., Au or Ag) that complicate integration; second, repeated sliding inevitably induces electrical wear and device degradation, making it challenging to simultaneously achieve high current densities and long‐term operational stability.

#### 3.2.3 Field‐Tunable Smart Interfacial Devices

Sliding interfaces in 2D nanomaterials can act as nanoscale “control knobs”, where external fields (electric, optical, or mechanical) actively and reversibly tune interlayer registry, thereby altering ferroic, electronic, or optical properties. Theoretical studies have predicted that interlayer sliding in systems such as BN and MoS_2_ can induce sizeable polarization, moderate switching barriers, and semiconducting characteristics, pointing to opportunities for non‐volatile memory; similarly, bilayers such as Cr_2_NO_2_ MXene or MoN_2_ exhibit strong magnetoelectric coupling, suggesting multifunctional potential [197]. Experimentally, Shalom et al. [198] realized out‐of‐plane ferroelectricity in twisted h‐BN bilayers (<0.5° misorientation) stabilized by sliding. Unlike conventional ionic‐displacement ferroelectrics, where oxygen‐vacancy migration (barrier ∼136 meV) causes fatigue and incomplete switching, sliding ferroelectrics localize charge defects at high‐energy barriers (2.6‐4.6 eV), thereby enabling fatigue‐free (>10^6^ cycles) polarization reversal, as demonstrated by Bian et al. [199] in bilayer 3R‐MoS_2_. Building on this, Yang et al. [54] fabricated 3R‐MoS_2_ heterostructures (Figure 6d), where Shockley partial dislocations serve as mobile domain boundaries; under electric fields, interlayer shear enabled robust ferroelectric switching with a 7 V memory window and > 10^4^ s retention, compatible with CMOS integration.

Beyond ferroelectricity, twist‐angle engineering allows continuous reconfiguration of moiré superlattices and their emergent properties. Ribeiro‐Palau et al. [55] demonstrated “twistable electronics” in BN/graphene/BN stacks (Figure 6e), where AFM‐controlled twisting modulated interlayer friction, electronic density of states, Raman signatures, and even valley Hall responses. Duan et al. [200] further exploited reconfigurable trilayer α‐MoO_3_ to create twistoptics systems with multiple optical “magic angles”, achieving on‐demand channeling of light across wide bandwidths and arbitrary in‐plane directions. Low‐friction sliding at metal/2D interfaces also modifies quantum confinement and edge‐state tunneling [201], while mechanical sliding of photonic or polaritonic crystals provides an alternative to conventional electro‐optic modulators that often suffer from high voltages and limited bandwidths [202]. For example, Xu et al. [203] constructed an air‐bridge NEMS device from a graphene‐Al‐based plasmonic grating (Figure 6f), where voltage‐controlled graphene‐metasurface spacing enabled deep modulation of ultraviolet plasmonic fields at sub‐150 mV bias and nanosecond (∼4 ns) response times. Yet, externally tunable devices face stringent requirements for integration and stability. Achieving atomic flatness, strain‐free alignment, and uniform doping is essential for robust sliding units, while dense integration of actuation, sensing, and readout modules at the micron scale requires fabrication processes that do not damage fragile van der Waals interfaces. Moreover, such devices are highly sensitive to vibrations, thermal fluctuations, and contamination, demanding specialized in situ packaging under high‐vacuum, low‐noise environments.

## 4 Emerging Directions and Challenges for 2D Superlubric Devices

While 2D superlubric materials have enabled prototype devices that transmit motion, harvest energy, and reconfigure interfaces, their practical implementation is constrained by three unresolved challenges. First, translating atomically smooth sliding into wafer‐scale devices requires reliable routes to transfer, stack, and integrate fragile nanomaterials without compromising their intrinsic properties. Second, the sheer diversity of nanomaterial combinations and interfacial configurations demands accelerated strategies that move beyond empirical trial‐and‐error toward data‐driven discovery and predictive design. Third, despite numerous proof‐of‐concept demonstrations, there is still no unified framework to benchmark device stability, durability, and compatibility with microsystem platforms, making cross‐comparison and industrial adoption difficult.

### 4.1 Scalable Transfer, Stacking, and System‐Level Integration

Direct synthesis of high‐quality stacked nanosheets on target substrates usually relies on chemical vapor deposition (CVD) [204, 205, 206] or gas/solid‐assisted CVD [207, 208] at elevated temperatures (>973 K). However, such high‐temperature processes often trigger undesirable covalent bonding with Si or other substrates [209, 210], compromising the interfacial integrity required for device performance. To circumvent this, transferring and stacking methods have emerged as effective solutions for constructing van der Waals interfaces with atomic‐scale precision [61, 62, 63]. For tribological applications, such approaches are particularly critical because an ideal superlubric interface must simultaneously satisfy two stringent conditions: (i) mobility with minimal energy dissipation, enabling sustained SSL or TSL, and (ii) stability to preserve mechanical, electronic, and optical functionalities under long‐term operation.

**FIGURE 7:**
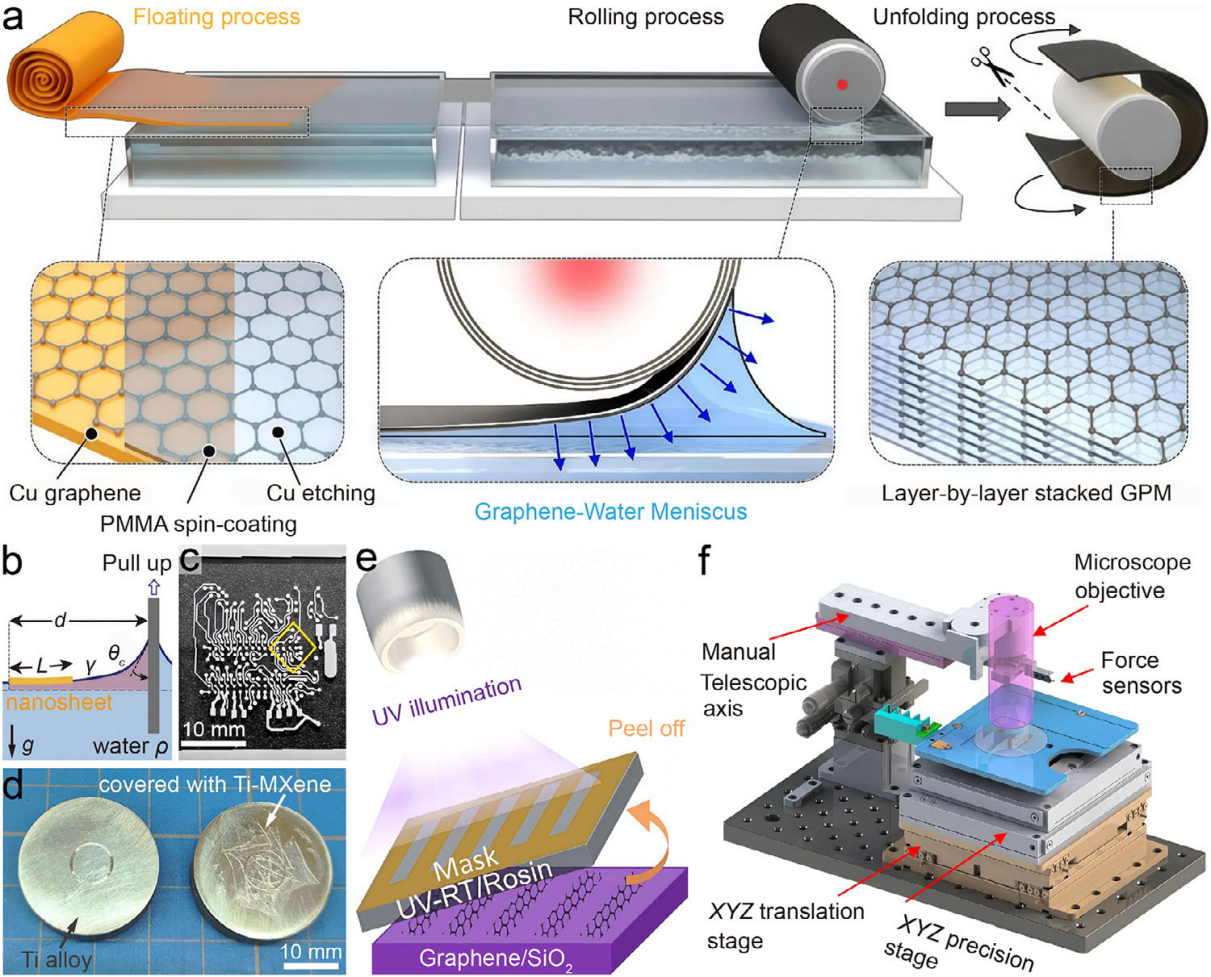
Transfer and integration approaches of 2D nanomaterials. (a) Schematic illustration of the float‐stacking process for a graphene‐PMMA laminate. Reproduced with permission from ref. [214]. Copyright 2024, Springer Nature. (b) 2D representation of the nanosheet capillary transfer mechanism. Reproduced with permission from ref. [215]. Copyright 2022, Wiley‐VCH. (c) Optical photographs of high‐entropy oxide scaffold nanomembranes fabricated on flexible circuits and Si wafers. Reproduced with permission from ref. [121]. Copyright 2025, Springer Nature. (d) Photographs comparing Ti alloy before and after coverage by Ti‐MXene nanomembranes following sliding at 473 K. Reproduced with permission from ref. [59]. Copyright 2025, Elsevier. (e) Roll‐to‐roll dry transfer and patterning of CVD graphene using ultraviolet‐release tapes as sacrificial layers. Reproduced with permission from ref. [236]. Copyright 2023, American Chemical Society. (f) Design schematic of the automated transfer and alignment system for wafer‐scale assembly. Reproduced with permission from ref. [240]. Copyright 2023, Springer Nature.

Sacrificial‐layer approaches (wet transfer) typically coat a polymer between the growth substrate and stacked nanosheets, dissolving it in liquid to detach the film with liquid support [211]. Poly(methyl methacrylate) (PMMA) has been widely used due to its solubility in organic solvents, enabling the transfer of graphene [212] and WS_2_ [213] nanosheets onto Si/SiO_2_ substrates. However, polymer‐assisted stacks, often hundreds of nanometers thick, are prone to wrinkles and trapped bubbles due to residual stress, adhesion mismatch, and capillary‐driven interfacial instabilities, which can accumulate during multilayer stacking. To overcome this, Kim et al. [214] exploited capillary flattening at the water‐air interface followed by roll‐to‐roll thermal pressing, achieving conformal and precise graphene transfer (Figure 7a). Park et al. [215]. further quantified the elasto‐capillary transfer process (Figure 7b), describing the critical distance $\left(d\;\leq \;\sqrt{\frac{\gamma }{\rho g}}\cdot ln\left(\frac{\gamma cos\theta_{c}}{\rho_{s}ghL}\right)\right)$ where spontaneous transfer occurs, determined by sheet density (ρ_*s*_), thickness (*h*), length (*L*), interfacial energy (γ), and equilibrium contact angle (θ_*c*_). For patterned transfer, polydimethyl s‐iloxane (PDMS) stamps provide a soft, low‐surface‐energy support, enabling precise placement of etched graphene onto circuits [216, 217]. Li et al. [121]. demonstrated an advanced example where a freestanding high‐entropy‐oxide scaffold was transferred via elasto‐capillary forces onto pre‐patterned microcircuits (Figure 7c), showing exceptional toughness (>300 MJ m^−3^), transparency (83.2%), adhesion, and oxidation resistance for stable encapsulation in harsh conditions. Despite versatility, PMMA and PDMS often leave cracks or residues that disrupt superlubricity [218, 219, 220, 221]. Replacing PMMA with paraffin yielded smoother graphene surfaces and cleaner interfaces [218], while polypropylene carbonate (PPC) enabled MoS_2_ nanosheets transfer with fewer residues and defects, suppressing increases in contact resistance and friction [222]. Alternative approaches avoid harsh etching altogether, such as directly growing 2D films on water‐soluble substrates like polyvinyl alcohol (PVA) gels [60, 120], or electrochemical delamination to precisely detach or regenerate substrates [223]. Furthermore, van der Waals assembly has proven to be a powerful strategy: Ti‐MXene nanomembranes can be conformally transferred and stacked onto titanium alloys (Figure 7d), acting as high‐temperature solid lubricants that reduce the COF and wear by 54% and 83% at 473 K, respectively [59].

Dry‐transfer strategies eliminate the involvement of liquids and solvents, mitigating contamination risks [224, 225]. Early approaches include thermally induced stress‐driven peeling, where mismatch in thermal expansion between layers drives controlled crack propagation [226], and elastomeric stamps (e.g., PDMS) that rely on tunable separation energy [227]. These methods enabled graphene transfer from Cu foils and MoS_2_ stacking on Si substrates [228, 229], though single‐stamp adhesion can distort fragile films. Protective polymer interlayers have been introduced to alleviate adhesion‐induced distortion [230, 231]. More advanced techniques exploit stimuli‐responsive adhesive layers: thermal release tapes (TRT) that decompose upon heating [232], and ultraviolet release tapes (UV‐RT) that undergo photochemical debonding with uniform force release [233, 234, 235]. Hung et al. [236] demonstrated a scalable UV‐RT roll‐to‐roll platform, enabling patterned, crack‐free graphene transfer with minimal residues (Figure 7e). For strongly bound epitaxial films, metal‐assisted transfer (Ni or Au, ∼140 meV/atom^−^^1^ adsorption) allows intact graphene peeling from SiC [237]. Matrix‐assisted approaches (using Au or SU‐8) offer directed interactions beyond van der Waals forces, enabling polymer‐ and solvent‐free transfer of monolayer MoS_2_ or graphene with high yield [238]. Inorganic‐membrane supports (e.g., metallized SiN_x_) further combine chemical inertness and flexibility for ultraclean assembly [239]. Recently, Chen et al. [240] developed a fully automated conveyance and alignment system (Figure 7f), leveraging atomically mismatched interfaces to suppress friction, enabling nearly energy‐free sliding during transfer. This method achieved wafer‐scale transfer with minimized cracks and precise positioning. Kong et al. [241] extended this concept to wafer‐scale van der Waals dry lamination, forming clean metal/2D semiconductor contacts with low resistance and high uniformity.

Overall, both wet and dry methods are evolving toward the same tribological imperative: constructing atomically clean, mechanically robust, and scalable van der Waals interfaces that preserve superlubricity during integration. Each technical refinement—whether capillary leveling, residue‐free supports, light‐activated release, or automated van der Waals assembly—directly addresses the stability of ultralow‐friction states, ensuring that transferred 2D nanomaterials can function reliably within MEMS/NEMS superlubric devices. Progress in this direction will be central to bridging the gap between laboratory demonstrations and reliable microsystem applications.

### 4.2 AI‐Guided Discovery and High‐Throughput Optimization

**FIGURE 8:**
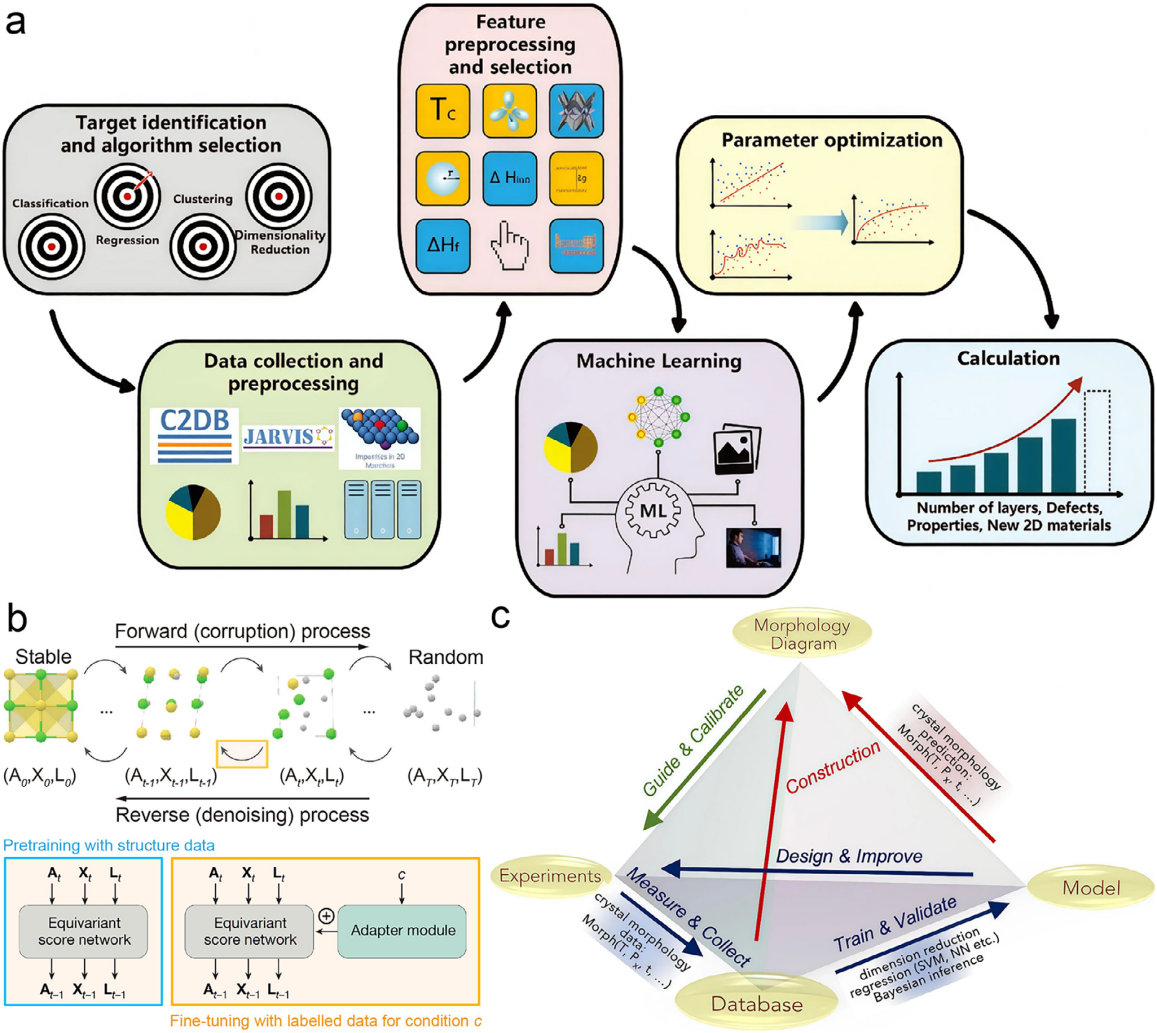
AI‐driven strategies for 2D nanomaterials design. (a) Reproduced with permission Typical workflow of ML model construction involving six steps, reorganized and redrawn based on ref. [65]. Copyright 2024, Wiley‐VCH. (b) Principle of the MatterGen framework for inverse design, where diffusion‐based reconstruction combined with adapter modules enables property‐controlled generation of stable materials. Reproduced with permission from ref. [250]. Copyright 2025, Springer Nature. (c) Materials Genome Initiative approach integrating experiments, modeling, and databases to establish morphology maps of 2D crystals. Reprinted with permission from ref. [268]. Copyright 2020, Elsevier.

The diversity of 2D nanomaterials, arising from stacking sequence, twist angle, and internal degrees of freedom such as spin, excitons, and sublattices, creates a vast design space that is both an opportunity and a challenge. Machine learning (ML) and high‐throughput (HT) computation have transformed the discovery of 2D materials with targeted static properties [64], but tribological performance for device‐grade 2D interfaces remains far less explored. This gap is central to the roadmap in Figure 1. Translating 2D‐enabled superlubricity into function‐ready microsystems requires discovery to be guided not only by lattice‐level mechanisms, but also by the constraints that set stability and lifetime at the device level. Accordingly, the relevant learning target is not a minimum COF in isolation, but a deployable performance state defined by operating windows, drift tolerance, cyclic durability, and integration compatibility. Figure 8a summarizes a conventional ML workflow for materials discovery, but tribological design requires each stage of that workflow to be reformulated around interfacial dynamics, environmental history, and device‐level constraints. Friction is an interfacial and dynamical response that spans multiple length scales. It is shaped by energy‐corrugation landscapes, defects, and environmental history, so models trained only on static descriptors are unlikely to generalize to device conditions. Most HT frameworks still rely on forward screening, in which large structural databases are generated and filtered by target properties [242]. In the tribology context, Tang et al. [243] recently formalized a geometry‐independent lubricating figure of merit (ζ) to enable HT ranking of low‐friction candidates, identifying more than 20 promising 2D crystals with potential lubricating performance superior to conventional layered materials. This result shows that HT descriptors can seed tribology‐oriented screening, but it also underscores the central limitation addressed in this section: static descriptors capture only part of the sliding dynamics and stability required under realistic constraints. Inverse design offers a complementary route by starting from desired performance and reconstructing plausible materials and interfaces [244]. With the evolution from genetic algorithms to deep neural networks [245], generative models such as variational autoencoders (VAEs) [246], generative adversarial networks (GANs) [247], diffusion models [248], and even large language models (LLMs) [249] have become capable of capturing complex distributions and introducing conditional constraints. For tribological design, this conditional capability is particularly important because it can target not only low sliding corrugation, optimal twist angles, or defect tolerance, but also the co‐constraints required for microsystem deployment, such as thermal stability, manufacturability, and, where interfaces act as functional layers, electrical or thermal transport. Figure 8b (MatterGen diffusion example) shows how joint diffusion over atom types, coordinates and lattice parameters can reconstruct stable crystals from noise while adapters enforce multi‐constraint control [250]. This is well aligned with the inherently multi‐objective nature of function‐ready tribological design.

Since 2018, ML has sparked extensive progress across 2D nanomaterials. Gaussian naive Bayes classification predicted 254 high‐moment 2D magnets [251]; Lasso regression screened 23,870 MXene bandgaps [252]; feed‐forward artificial neural networks modeled atomic layer deposition kinetics [253]; support vector machines and deep neural networks uncovered exfoliation energies and structural stability of layered systems [254, 255]; and random forest algorithms optimized wafer‐scale growth of 2D semiconductors [256]. Although these studies target different endpoints, together they establish a toolkit that is directly relevant to the engineering axis in Figure 1, because scalable synthesis, interface formation, cross‐wafer uniformity, and integration yield ultimately determine whether a superlubric interface can be transferred, patterned, and stabilized at the microsystem level. Coupled with DFT and MD, ML has accelerated discovery of mechanical [257, 258, 259], electrical [260, 261], and thermal [262, 263] properties by learning reliable descriptors and scaling to large datasets. Yet predictive models for tribological properties remain scarce. A central obstacle lies in the lack of dynamical tribological labels such as sliding barriers, interfacial corrugation maps, shear strength or wear‐rate trajectories. Recent tribology reviews emphasize that nanoscale force spectroscopy, particularly AFM‐based techniques, provides a systematic pathway to obtain such dynamical labels by quantifying adhesion, shear strength, and interfacial dissipation at the atomic level [264]. Embedding these measurements into ML frameworks is one practical route to bridge static calculations and dynamic sliding responses relevant to superlubricity. This requires descriptors and standardized metadata that are explicitly tribology‐aware. Relevant quantities include adhesion and exfoliation energy, DFT‐ or MD‐derived sliding barriers, contact‐pressure descriptors, bending stiffness, and defect formation energies. Equally important are device‐level metadata, including load or contact stress, sliding velocity and pv, temperature, and humidity or atmosphere history. These quantities should be paired with computational, AFM, and tribometer protocols that enable interoperable datasets. Models should be physics‐aware and uncertainty‐aware. Physics‐ or symmetry‐aware graph representations introduce inductive bias that improves generalization and physical consistency, particularly when extrapolating across materials and operating conditions. Uncertainty quantification (UQ) helps identify high‐uncertainty or out‐of‐distribution regimes and therefore reduces the risk of overconfident, unphysical interpretation. Active learning can then improve data efficiency by prioritizing new measurements or simulations, with performance depending on the chosen acquisition strategy. This perspective resonates with the broader “tribo‐informatics” framework, which calls for structuring friction and wear data into interoperable formats and coupling statistical learning with mechanistic insight [265]. In this sense, the microsystem benchmarks introduced in Section 2.3, including pv windows, environmental history, cyclic durability, and failure‐mode definitions, should be treated not only as reporting items but also as metadata fields and learning targets.

Incorporating tribo‐informatics principles is essential if AI‐driven tribology is to move beyond fragmented case studies toward standardized, reusable datasets and closed‐loop predictive workflows. Deep generative models, in turn, open a route to inverse design of 2D interfaces with multiple objectives. In practice, superlubricity cannot be optimized in isolation but must be balanced with device‐relevant requirements such as electrical conductivity, thermal stability, and manufacturability. Conditional VAEs, GANs, and diffusion models are particularly attractive in this context because they can enforce such coupled constraints during generation, placing tribology on equal footing with other interface functions. Recent work on amorphous solids [266], probabilistic crystal generation [267], and deep generative inverse design [245, 250] shows that stable structure generation under multiple constraints is feasible, although ensuring robustness under cycling and environmental perturbations remains a key challenge. A complete discovery loop will also require automated transfer and stacking methods together with HT tribometry, so that experimental outputs can continuously update models in real time. A coordinated synthesis‐measurement map, analogous to the Materials Genome Initiative's growth‐morphology maps shown in Figure 8c [268], provides a practical blueprint for translating predictions into manufacturable superlubric devices. In short, AI will be effective for superlubric 2D microdevices only if the community (i) builds tribology‐specific descriptors and standardized datasets, (ii) adopts physics‐informed, UQ‐equipped ML and conditional generative models for multi‐objective design, and (iii) integrates these models with automated transfer and tribological testing to form closed‐loop discovery. Only then can ML move from predicting static materials properties to reliably engineering interfaces that sustain ultralow friction under realistic device constraints.

### 4.3 From Mechanisms to Metrics: Standardizing 2D Superlubric Interfaces

Although AI‐assisted discovery has expanded the library of candidate 2D superlubric interfaces, tribological evaluation across length scales remains fragmented and often difficult to compare, because reported metrics are not consistently linked to operating windows, interfacial state, and failure modes. To move toward function‐ready microsystems, this section establishes a mechanism‐to‐metrics translation that maps nanoscale observables to device‐relevant performance descriptors, enabling consistent interpretation from idealized incommensurate contacts to tribofilm‐governed engineering tests and integration‐ready interfaces.

#### 4.3.1 Micro/Nanoscale Evaluation of 2D Superlubric Interfaces

**FIGURE 9:**
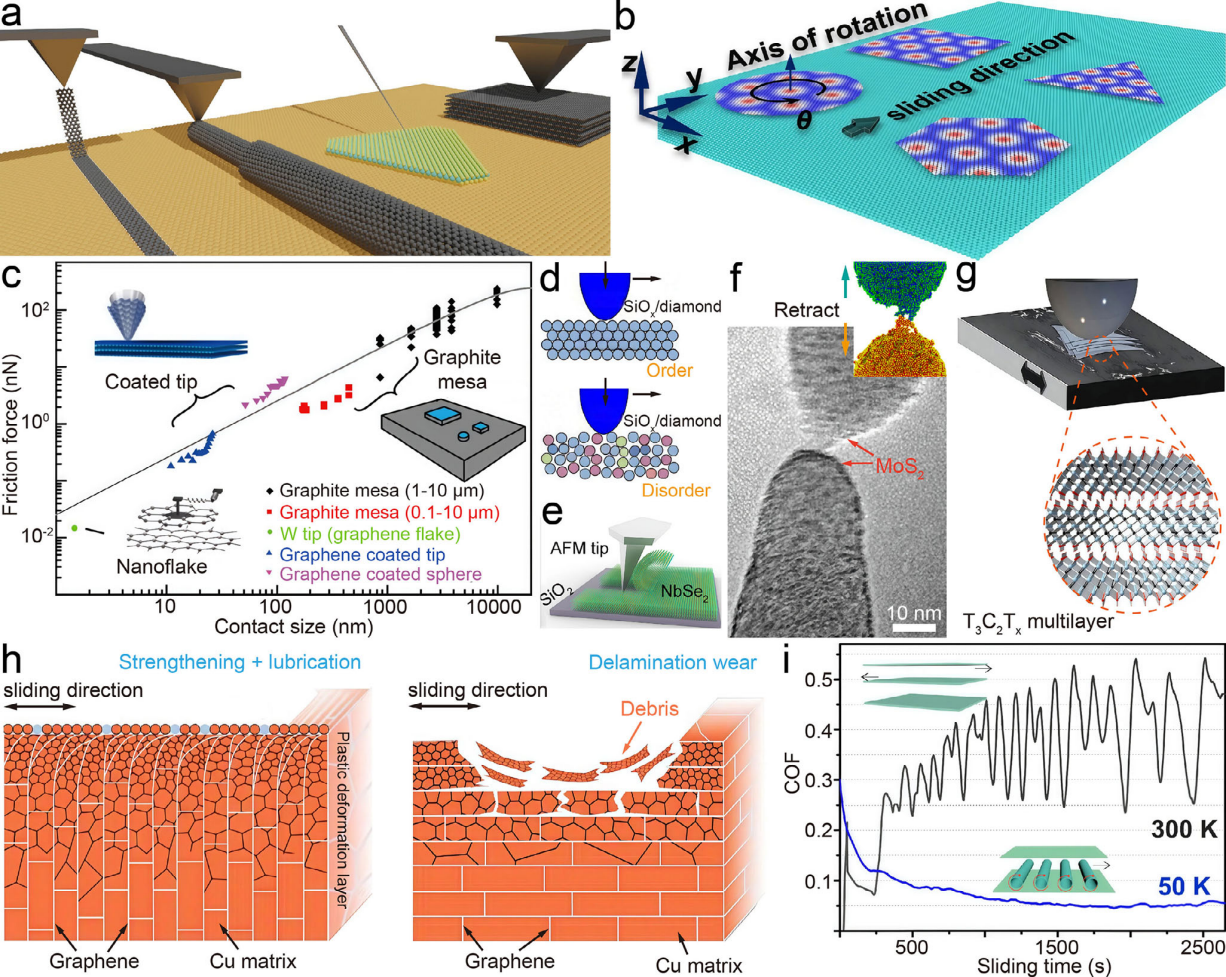
Multiscale evaluation of superlubric 2D interfaces. (a) Typical manipulations used in microscale experiments to probe 2D superlubric interfaces. Reproduced from ref. [88]. Copyright 2024, American Physical Society. (b) Sliding of graphene flakes with varied contact shapes on a fixed graphene substrate (twist angle = 5°). Reproduced from ref. [273]. Copyright 2024, Elsevier. (c) Friction‐size relation of square graphite mesas (0.8–10 µm). Reproduced from ref. [279]. Copyright 2020, American Physical Society. (d) Comparative schematic of SiO_x_/diamond tips sliding on crystalline Pt and Pt‐based amorphous alloys. Reproduced from ref. [280]. Copyright 2013, American Chemical Society. (e) Distinct tribological responses of mono‐ and multilayer NbSe_2_ on SiO_2_ substrates. Reproduced from ref. [282]. Copyright 2023, American Chemical Society. (f) In situ TEM image of MoS_2_‐coated Si tips in contact and separation; inset: atomistic simulation of MoS_2_ adhesion. Reproduced from ref. [288]. Copyright 2024, American Chemical Society. (g) Enhanced wear resistance of multilayer Ti_3_C_2_T_x_ MXene film on steel/Si_3_N_4_ interface. Reproduced from ref. [295]. Copyright 2021, American Chemical Society. (h) Orientation‐dependent friction of graphene/Cu composites. Reproduced from ref. [298]. Copyright 2021, Elsevier. (i) Cryogenic temperature molecular rolling lubrication of graphene by spontaneous edge curling. Reproduced from ref. [104]. Copyright 2024, Springer Nature.

Understanding and standardizing superlubricity at the micro/nanoscale is central to translating 2D interfaces into frictionless components for MEMS/NEMS. Yet, as contact dimensions increase, the scaling interpretation of SSL and potential TSL becomes ambiguous, defying direct extrapolation to device‐scale behavior. In classical dry friction, the friction force is often approximated as *F*  ≈  µ*N*, where *F* is the friction force, *N* is the normal load, and µ is the COF. In the Bowden‐Tabor picture, *F*  ≈  τ*A_r_*, where τ is an effective interfacial shear strength and *A_r_* is the real contact area, which in many contact models increases with *N* [269]; this provides a practical basis to discuss friction‐area scaling under specified loading conditions. For atomically flat, incommensurate 2D interfaces, the friction force can instead follow a sublinear power law *F*_*s*/*k*_ ∝ *A* ^γ^ with scaling exponent γ  <  1, where *F*_*s*/*k*_ denotes the static/kinetic friction force and *A* is the contact area, reflecting partial cancellation of lateral forces during sliding [270]. Figure 9a summarizes several AFM and micro‐manipulation experiments probing 2D superlubric contacts, conditions analogous to microsystem operation, where a constant COF $\left(\frac{F_{s/k}}{N}\right)$ under fixed normal load (*N*) marks the onset of superlubricity. Tosatti et al. [88] emphasized that current micro‐scale experiments still lack unified standards: discrepancies persist in the scaling of friction versus area (from sublinear to linear), the critical balance between edge pinning and interior sliding, and the weak velocity dependence observed experimentally (logarithmic rather than linear) contradicts theoretical predictions. Moreover, the real COF under strong adhesion and finite load remains ill‐defined, and the role of defects, steps, and adsorbates in disrupting superlubricity is often overlooked. For cross‐study comparability, micro/nanoscale reports should explicitly specify the fitting range for 𝐴 and 𝑁 (or contact stress), the extracted γ, the interfacial state (defects/steps/adsorbates), and the operational window over which a superlubric state is sustained.

Beyond size scaling, the geometry of contact critically modulates frictional behavior. For instance, irregular Au nanocrystals sliding on graphite exhibit γ  ≈  0.25 or 0.75 [271], while twisted bilayer graphene displays *γ* = 0.5 for circular and *γ* = 1 for hexagonal contacts [272]. Yan et al. [273] further demonstrated that in twisted layered interfaces, the scaling exponent is fundamentally governed by the slider geometry and its edge alignment with the moiré superlattice (Figure 9b). Circular flakes, where edge compensation is incomplete, follow *A* ^1/4^; polygonal flakes misaligned with the moiré lattice show oscillatory yet scale‐independent behavior (γ  ≈  0); whereas aligned polygonal edges yield *A* ^1/2^ scaling. Based on the 2D Frenkel‐Kontorova model, Mandelli et al. [274] proved that both static friction and scaling exponents are extrinsic, strongly dependent on the driving mode. For Au/graphite heterojunctions, edge pulling induces local commensuration and enhances friction, whereas edge pushing preserves incommensurability and maintains ultralow friction. Beyond crystalline interfaces, amorphous contacts also exhibit sublinear scaling (γ  ≈  0.5), but contamination can restore Amontons‐like linearity [275]. Local roughness, substrate corrugation, and lattice registry—highly temperature‐sensitive—govern static friction; for instance, krypton islands on Pb(111) show γ≈0.25∼0.37 due to edge pinning [276]. As the contact area increases, various dissipative modes (as shown in Figure 2a) inevitably drive friction from sublinear to linear scaling [277, 278]. AFM tests on graphene interfaces (Figure 9c) reveal near‐linear scaling of total friction with contact size below 10 µm due to edge‐dominated friction, which evolves toward quadratic dependence as internal atomic interactions dominate [279]. Accordingly, γ should not be treated as a materials constant but reported together with slider geometry, edge‐moiré alignment, driving mode, and contamination control/cleaning history, which collectively determine whether sublinear scaling persists.

Superlubricity at this scale is also time‐dependent, sensitive to tip wear, velocity, and load [35]. In single‐asperity AFM contacts, chemical and structural factors jointly determine friction and wear. As shown in Figure 9d, with a chemically active SiO_x_ tip sliding on Pt surfaces, crystalline Pt(111) exhibits a higher COF (∼0.06) than Pt‐based metallic glass (∼0.03) due to tribo‐induced interfacial alloying; in contrast, chemically inert diamond tips yield ultralow COF (∼0.002) on both surfaces [280]. Such comparisons underline the necessity of standardizing tip chemistry and interfacial reactivity to obtain reproducible metrics. Similarly, in MEMS sensors, controlled modulation of interfacial chemistry can tune periodic frictional responses, as interfacial “reconstruction” reshapes the potential‐energy landscape [281]. The structural order and substrate coupling of 2D layers add further complexity. For instance, Figure 9e shows that monolayer NbSe_2_ on SiO_2_ exhibits stronger adhesion and charge transfer than multilayers, effectively suppressing out‐of‐plane wrinkling and achieving a stable ultralow COF (0.0085), while multilayer stacking enhances friction and wear [282]. Scanning Kelvin probe microscopy further indicates that high‐stress pre‐rubbing can increase the work function of graphene on SiO_2_/Si substrate, consistent with interfacial electron transfer that modifies adhesion and contact stiffness and correlates with reduced friction [283]. Even when charge transfer between the two contacting solids is weak, adhesion can remain strongly substrate‐mediated, because non‐additive van der Waals interactions in tip/2D/substrate stacks can measurably reshape the adhesion landscape (e.g., graphene on Cu) [284]. Importantly, van der Waals adhesion at 2D heterointerfaces can also be quantified through normalized critical adhesion pressure using graphite‐based probes, providing a practical, comparable adhesion metric rather than qualitative “sticky/clean” descriptors [285]. In parallel, local chemical affinity can shift the balance between adhesion‐controlled and shear‐controlled frictional responses at metal interfaces [281]. Environmental water and surface chemistry provide another independent lever, as hydrophilicity‐dependent MXene interfaces show humidity‐sensitive adhesion/friction coupling [33]. At minimum, reports should state the tip material/termination and reactivity class, tip conditioning and wear state, pre‐sliding history, environmental history, and an adhesion/contact‐quality metric. Where charge transfer or electrical functionality matters, also report an electronic‐state descriptor (e.g., work‐function shift or contact‐potential difference).

Advanced in situ methods are now bridging the mechanistic gap between atomic‐scale modeling and MEMS‐scale evaluation. High‐resolution TEM‐AFM platforms allow direct visualization of discrete stick‐slip dynamics [286] and diffusion‐mediated liquid‐like sliding beyond the classical Prandtl‐Tomlinson model [287]. As shown in Figure 9f, Toom et al. [288] combined in situ TEM‐AFM with MD to show that reducing grain boundaries in MoS_2_ nanosheets deposited on Si tip pair suppresses covalent bond formation across the contact, lowering adhesion energy and material transfer. These findings demonstrate that quantitative, mechanism‐linked observables, such as scaling exponent, critical shear stress, sliding‐barrier energy corrugation, work‐function shift, and lifetime of the superlubric state, should form the core of standardized reporting. To enable reproducible translation, these observables should be reported as a core set with consistent definitions, measurement protocols, and operating‐window metadata, so that COF and wear can be interpreted in their microstructural and chemical context rather than as standalone numbers. Establishing mechanism‐based, parameterized standards, where each COF or wear rate is linked to its microstructural and chemical context, is essential to bridge AFM‐scale understanding with MEMS‐level implementation. Such standardization will enable reproducible evaluation of 2D superlubricity, turning phenomenological observations into quantifiable engineering metrics.

#### 4.3.2 Macroscale Evaluation and Tribofilm‐Based Standards

The tribological evaluation of 2D nanomaterials at the engineering scale commonly relies on ball/pin‐on‐disk and reciprocating configurations, which are widely used as engineering‐relevant screening configurations for practical sliding contacts [289, 290]. In most cases, 2D nanomaterials act as lubricating or reinforcing phases, either deposited as coatings or embedded into composites, where dynamically formed tribofilms typically dominate frictional response [291, 292]. However, the measured COF and wear rate in such tests inherently reflect the mechanical and chemical stability of dynamically generated tribofilms, and thus cannot be interpreted as a direct proxy for the intrinsic lattice‐level properties of 2D nanomaterials such as their ideal shear strength or van der Waals sliding potential. This scale paradox, between nanoscale physics and macroscale engineering outcomes, is the core reason behind the current lack of unified evaluation standards. Despite this challenge, the incorporation of ultralow‐friction 2D interfaces into macroscale systems has yielded remarkable improvements. For example, 2 wt.% graphene in polyimide improves wear resistance twentyfold and decreases COF by 12% [293]; 1 wt.% TiO_2_/Ti_3_C_2_T_x_ hybrid nanocomposites reduce the COF of stainless steel in oil lubrication to 0.073 [294]. Compared with graphene and MoS_2_, MXenes possess stronger interlayer bonding, reducing catastrophic delamination. As shown in Figure 9g, a multilayer Ti_3_C_2_T_x_ tribofilm lowers COF sixfold under 300 MPa contact pressure, reaching a wear rate of 4 × 10^−9^ mm^3^/Nm, outperforming MoS_2_ and graphene coatings by two orders of magnitude [295]. During sliding, MXene nanosheets undergo thermo‐mechanical degradation into amorphous or oxide‐rich tribolayers that lower shear resistance [139, 296]. Synergistic interfaces such as Ti_3_C_2_T_x_‐GO further enhance adhesion and compactness, mitigating the delamination commonly seen in single‐component coatings [297]. For meaningful comparison at this scale, COF and wear should be interpreted together with tribofilm state variables, at minimum including tribofilm continuity and thickness, phase/chemistry, and preferred orientation, because these govern whether low shear is sustained or lost during sliding.

However, such macroscopic data are often non‐unique and challenging to reproduce across laboratories. Friction and wear results are highly sensitive to sliding direction, contact geometry, humidity, and even substrate crystallography. For instance, Figure 9h shows that in graphene/Cu composites with a “brick‐and‐mortar” laminated structure, sliding perpendicular to graphene edges yields a 23% lower COF and tenfold better wear resistance than pure Cu, while parallel sliding triggers delamination and catastrophic wear [298]. Dynamic in situ formation of 2D superlubric interfaces offers a possible route to mitigate such anisotropy: in FeCoCrNiAl high‐entropy alloy composites, friction‐induced shearing of graphite nanoparticles produces graphene nanosheets in tribofilms, reducing COF by up to 80% and wear rate by nearly two orders of magnitude [299]. For MEMS/NEMS applications, however, the protective coatings must remain ultrathin while maintaining both low friction and wear resistance. Introducing metal‐ion bridges between 2D interfaces to form “brick‐concrete” architectures effectively enhances cohesion without increasing thickness, offering a practical route for silicon‐based microdevices [300]. Temperature effects add another dimension to tribological evaluation. At 50 K, graphene edges spontaneously curl to form molecular‐bearing‐like rolling contacts, reducing COF from 0.25–0.45 to 0.04–0.06 (Figure 9i); in DFT simulations of the corresponding model junction under the same load (7.9 GPa), the maximum friction potential‐energy variation along the rolling pathway (5.1 meV, for the simulated interface model/supercell) is far smaller than that of sliding (146.4 meV) [104]. While these results demonstrate the versatility of 2D nanomaterials in macroscale lubrication, they also expose the non‐reproducibility and non‐standardization of existing methods. Current protocols cannot resolve tribofilm nucleation, growth, and failure in real time, effectively reducing macroscale measurements to a “black box” in which the time‐dependent pathways that set friction and wear are not recorded. Environmental parameters such as humidity, temperature, and ambient gas are rarely quantified or standardized, preventing any meaningful cross‐laboratory comparison. Accordingly, reports should specify the full test geometry and counterface, sliding direction relative to microstructure/texture, and the time history of key environmental variables (humidity, temperature, and ambient gas), together with any run‐in or conditioning history that governs tribofilm evolution.

To move beyond empirical comparisons, tribological evaluation of 2D nanomaterials must transition from COF reporting to a mechanism‐driven, multiscale standardization paradigm. Current macroscale tests suffer from non‐reproducibility because they measure the dynamic outcome of tribofilm evolution rather than its formation mechanisms, and often neglect precise control of environmental variables. As a result, COF values across laboratories remain incomparable and detached from microsystem operating conditions. Standardization should thus be anchored in three pillars: (i) micro/nanoload quantification, incorporating low‐load, high‐frequency regimes relevant to MEMS; (ii) graded durability metrics, defining “tribological robustness” as COF stability within humidity or temperature thresholds; and (iii) mandatory mechanistic validation, requiring quantitative analysis of tribofilm thickness, phase composition, and lattice orientation. Such requirements would bridge nanoscale interfacial physics with macroscale engineering metrics. In practice, this means reporting a minimal, mechanism‐linked metadata set that couples loading and kinematics (including contact model and pv window), environmental history and durability criteria (drift rate and failure definition), and tribofilm structure (thickness/continuity, phase/chemistry, and orientation supported by quantitative characterization). With this interoperable schema, macroscale tribology data can move from isolated COF values to transferable, integration‐relevant evidence, enabling predictive screening and iterative optimization of scalable 2D superlubric interfaces.

## 5 Outlook

Over the past decade, the study of superlubricity in 2D nanomaterials has evolved from a conceptual understanding of frictionless motion into a design‐driven science of controllable interfacial energy dissipation. Fundamental insights into structural and phase‐transformation superlubricity have clarified how lattice incommensurability, twist angle, and interfacial reconstruction govern the transition between pinned and sliding states. These microscopic mechanisms have reframed friction not as an inevitable loss but as a tunable parameter in low‐dimensional systems, bridging the gap between atomic‐scale physics and macroscale tribological behavior. Moving from principles to practice, recent progress in van der Waals assembly, layer transfer, and scalable stacking has enabled function‐oriented integration of 2D nanomaterials into microsystems. Such advances underline a paradigm shift—from passive friction reduction to the programmable control of mechanical, electrical, and thermal coupling at buried interfaces. Yet, translating atomic superlubricity into device‐level reliability remains an unresolved challenge, constrained by contact variability, environmental sensitivity, and scale‐dependent instabilities.

Looking forward, achieving predictable and standardized superlubricity will require a convergence of multiscale characterization, data‐centric modeling, and mechanism‐enforced testing. Artificial intelligence and automated tribometry promise to bridge static computation and dynamic experiments, enabling closed‐loop discovery of 2D interfaces optimized for energy dissipation and durability. Meanwhile, a unified, mechanism‐based evaluation framework is urgently needed, one that links nanoscopic descriptors (e.g. corrugation energy, interlayer shear strength) with macroscopic metrics (COF, wear rate) under well‐defined micro/nano contact conditions. The goal extends beyond minimizing friction: it is to architect adaptive, energy‐programmable 2D interfaces capable of sustaining stability and functionality across scales. Such a shift, from understanding superlubricity to engineering it, will define the next frontier of nanoscale tribology and its integration into future intelligent microdevices.

## Conflicts of Interest

The authors declare no conflicts of interest.

## Data Availability

The data that support the findings of this study are available from the corresponding author upon reasonable request.
